# A Critical Review of the Neuropharmacological Effects of Kratom: An Insight from the Functional Array of Identified Natural Compounds

**DOI:** 10.3390/molecules28217372

**Published:** 2023-10-31

**Authors:** Rahni Hossain, Abida Sultana, Manit Nuinoon, Kunwadee Noonong, Jitbanjong Tangpong, Kazi Helal Hossain, Md Atiar Rahman

**Affiliations:** 1 School of Allied Health Sciences, College of Graduate Studies, Walailak University, Nakhon Si Thammarat 80160, Thailand; rahni.ho@mail.wu.ac.th (R.H.); manit.nu@wu.ac.th (M.N.); kunwadee.no@wu.ac.th (K.N.); 2 Department of Biochemistry & Molecular Biology, University of Chittagong, Chittagong 4331, Bangladesh; adibaabida800@gmail.com; 3 Hematology and Transfusion Science Research Center, Walailak University, Nakhon Si Thammarat 80160, Thailand; 4 Research Excellence Center for Innovation and Health Product (RECIHP), Walailak University, Nakhon Si Thammarat 80160, Thailand; 5 Angiogenesis and Brain Development Laboratory, Department of Neurosciences, Huntington Medical Research Institutes (HMRI), Pasadena, CA 91105, USA; kazi.helal@hmri.org

**Keywords:** kratom, neurological effects, mitragynine, 7-Hydroxymitragynine, antioxidant, anti-inflammation

## Abstract

Kratom (*Mitragyna speciosa* Korth. Havil) has been considered a narcotic drug for years, barred by the law in many parts of the world, while extensive research over the past few decades proves its several beneficial effects, some of which are still in ambiguity. In many countries, including Thailand, the indiscriminate use and abuse of kratom have led to the loss of life. Nonetheless, researchers have isolated almost fifty pure compounds from kratom, most of which are alkaloids. The most prevalent compounds, mitragynine and 7-hydroxy mitragynine, are reported to display agonist morphine-like effects on human μ-opioid receptors and antagonists at κ- and δ-opioid receptors with multimodal effects at other central receptors. Mitragynine is also credited to be one of the modulatory molecules for the Keap1-Nrf2 pathway and SOD, CAT, GST, and associated genes’ upregulatory cascades, leading it to play a pivotal role in neuroprotective actions while evidently causing neuronal disorders at high doses. Additionally, its anti-inflammatory, antioxidative, antibacterial, and gastroprotective effects are well-cited. In this context, this review focuses on the research gap to resolve ambiguities about the neuronal effects of kratom and demonstrate its prospects as a therapeutic target for neurological disorders associated with other pharmacological effects.

## 1. Introduction

A variety of natural products have been used as medicines and have been associated with traditional medicine for thousands of years [1]. Compounds derived from natural products have gained substantial market share or serve as biochemical tools for demonstrating the role of specific pathways in disease and their potential as drugs [2]. Thus, natural products have historically consisted of the most successful sources of new medicines. Compounds derived from natural products serve as both drugs and templates for drugs directly, as well as leading to the discovery of novel aspects of physiology or biology that assist in better understanding disease targets and pathways [1,3]. According to the World Health Organization, natural products are considered to be important sources of medicine because of their traditional uses or remedies, and through a few systematic approaches to exploring naturally used products or compounds that can be developed as drug leads, the world has recognized the importance of natural products as medicines [4]. To find drugs from natural products, it is important to know which active compounds accurately target the pathways of disorders.

An indigenous Southeast Asian plant with specific medicinal benefits, *Mitragyna speciosa* Korth. (Rubiaceae) Havil. is also known as kratom, kakuam, ithang, thom in Thailand; ketum or biak-biak in Malaysia; or krypton when combined with O-demethyltramado [5,6]. Thailand, Indonesia, Malaysia, Myanmar, and Papua New Guinea are among the countries that originated it [7,8]. Different formulations are available for kratom, including raw leaves, tea, capsules, tablets, powders, and concentrated extracts. It is widely used for anxiety, depression, pain relievers, talkativeness, sociability, sedation, mood enhancement, constipation, increase energy, appetite, sexual desire, wound healer as a local anesthetic, and other ailments/conditions as a traditional medicine. The Ketum solution is recommended to ease opiate withdrawal symptoms by taking three 3 × 250 mL daily [9,10,11,12]. Due to addiction concerns and an increase in the number of young people utilizing the leaf material and developing a “hook”, kratom was formerly outlawed in Thailand and the adjacent country of Malaysia [13]. By using a quick and reliable PCR-reverse dot blot (RDB) hybridization experiment, kratom demonstrated the presence of several narcotic specimens, such as ground leaves and kratom’s cocktail, particularly in matK sequences, a diagnostic barcode [14]. In a different investigation, DNA barcoding in conjunction with high-resolution melting (Bar-HRM) analysis was used to confirm the validity of kratom as a species of narcotic plant for law enforcement [15]. Since at least the eighteenth century, it has been employed in herbal medicine, with various therapeutic benefits, including antioxidant [16,17,18], antimicrobial, antibacterial [16,19], antinociceptive [20], anti-inflammatory [21], cytotoxic [20], weight reduction [22], analgesic [23,24,25,26], antipyretic, sedative, stimulant, antidiabetic, anxiolytic and anti-depressant [27,28,29,30,31,32], antidopaminergic [33], and antidiarrheal. Very recently, Salleh et al. reported the potential neuroprotective role of kratom on aging brains [34]. Zul Aznal et al. have shown that mitragynine, the major compound of kratom, exposure in adolescent animal brains causes deficits in social behavior but cognitive behavior remains unaffected [35]. Within this controversy, Singh and his colleagues proved that a higher intake of kratom juice (>3 glasses daily) did not appear to impair the motor, memory, attention, or executive function of regular kratom users [36]. In this context, this review dissects the neurological role of kratom and its phytoconstituents to bridge the research gap and reveal whether kratom could be used in neurological disorders by sketching out the underlying molecular mechanisms and relevant pharmacological action.

## 2. Methodology

In this review, the literature search was conducted using PubMed, Google Scholar, and Scopus from June 2010 to July 2022 to retrieve information about consumption, health effects, phytochemistry, phytoconstituent, toxicology, pharmacokinetics, case reports, and pharmacological effects of kratom using the keywords antioxidant, anti-inflammatory, neuroprotective, antidiabetic, anxiolytic, and anti-depressant. Additionally, a literature search was performed to find pertinent human and animal studies. Various research papers, reviews of the literature, and case studies on kratom were also included in this review. 

## 3. Focus of This Review

**Scheme 1:**
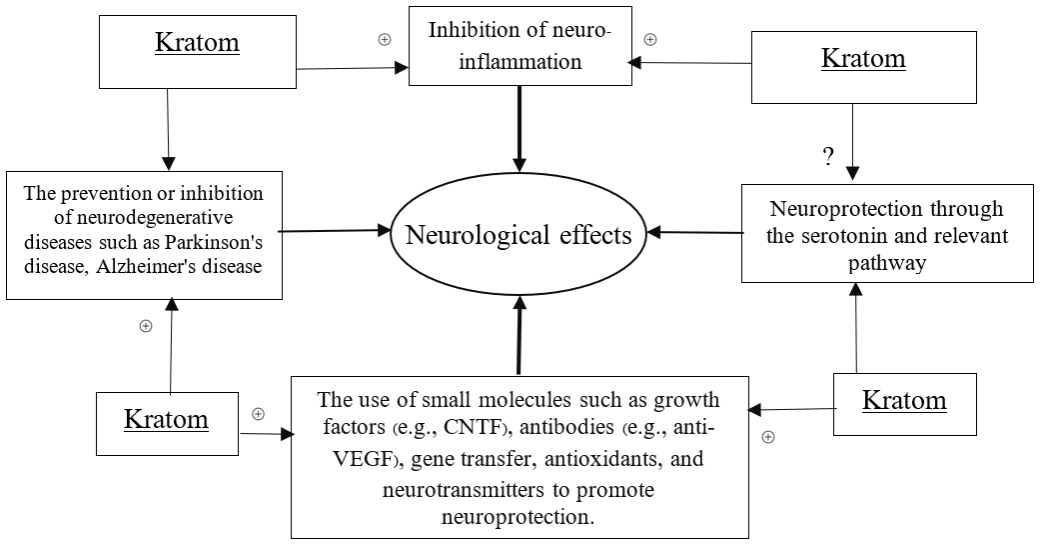
Schematic diagram to define neurological effects. (+) sign indicates the positive modulatory effects of Kratom in neuroprotection through the proposed scheme.

Kratom has been known to create addiction for many years. However, over the past years, scientists’ extensive research on kratom has re-introduced it to people as a wonder. The CNS effects of kratom became a precious context of drug exploration. Almost all the compounds analyzed in kratom are important alkaloids or derivatives [37]. So far, research findings on the major compound of kratom, mitragynine, suggest its mixed and controversial effect on cognitive behavior, while some other researchers reported the neuroprotective effect of kratom, although the true mysteries remain unknown. However, by combining the efficacy of kratom’s alkaloids, especially the mitragynine and 7-hydroxy mitragynine, with those demonstrating neurological activity, we designed the necessary approaches and models to uncover its neurological potential. We specifically sought to propose the mechanisms of how kratom can potentially contribute to neurological properties through the linked biochemical and pharmacological actions including antioxidative, anti-inflammatory, and related gene modulatory functions. A schematic diagram has been placed to hypothesize the neurological effects of kratom (Scheme 1).

## 4. Nomenclature and Provenance

**Figure 1:**
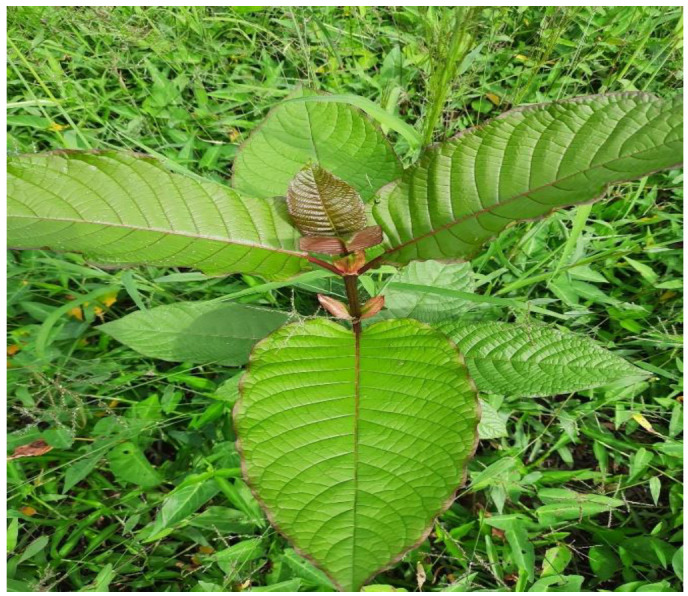
The specimen of the kratom plant from the Walailak University campus in Thailand.

A notable example of a new psychoactive drug (NPS) of natural origin is kratom (Korth.) Havil (Figure 1), a tropical tree that may reach heights of 4 to 16 m and is found in both Asia and Africa [13]. In various regions of Southeast Asia, manual laborers have traditionally made tea or chewed on the tree’s chopped fresh or dried leaves to reduce weariness and boost productivity [38]. Kratom compounds have also been used for thousands of years in ceremonial social rites and to cure a wide range of diseases, including opium addiction in Malaya and morphine dependence in Thailand [39].

The name “*Mitragyna*” for the genus is thought to have been given by the Dutch botanist Korthals because the stigmas and leaves of the plant’s flowers have the same shape as a bishop’s miter [40]. Though it might be inferred that the word derives from “Mithraic cults” which have long been regarded as a source of spiritual transcendence, given its broad variety of applications [41]. The kratom tree, which is utilized as an alternative to alcohol and opium in Thailand, is primarily found in the southern part of the nation and is easily bought from teashops. Two different types of kratom can be distinguished by the color of the leaf vein, which can be either red or green. The crimson vein, which is renowned for its abrasiveness and lasting effects, is often preferred by the locals [13]. Although fresh leaves are often chewed and taken as a powder at a dosage of 10 to 30 fresh leaves per day, they can be smoked or used to make tea [42].

The Kratom Act was passed in Thailand in 1943, and it was thought that this action was taken more for economic reasons than out of concern for the general welfare of the population. Taxes were levied on the opium trade at the time, and since this was so expensive, people began switching to kratom as a replacement, which had an impact on the Thai government’s revenue. The Thai government categorized kratom under Category V of a narcotics categorization in the Narcotics Act later in 1979, among cannabis, opium, and hallucinogenic mushrooms (the least restrictive and punitive level) [42,43,44].

Kratom has been on the Poison Schedule List since the 1952 passage of the Poison Act. According to the First and Third Schedules of Malaysia’s Poison Act 1952, which was amended in 2003, mitragynine, which is found in kratom leaves, is toxic. As a result of Section 30 (3) of the Poison Act of 1952, anyone who violates subsection (3) or any regulations under this Act pertaining to psychotropic substances faces a fine of up to ten thousand ringgit or imprisonment for up to four years. Using kratom improperly can result in a 4-year jail sentence or fines not exceeding RM 10,000 or both [45].

In the beginning, kratom was mostly utilized for its therapeutic benefits in treating minor medical issues like fever, diarrhea, diabetes, and discomfort as a wound poultice, as well as to alleviate the strain and tiredness of physical labor. But because it is readily available and inexpensive, it has also gained recognition for its use in the suppression of opiate withdrawal symptoms [9,43,44,46]. Kratom tea has recently become a popular base for the “4 × 100” drink, which combines Coca-Cola, cough syrup, ice cubes, and kratom tea. This eventually became a concern because these users were enhancing the effects with substances such as benzodiazepines [42]. A study assessed the cognitive function of 70 regular kratom users and 25 control participants using the Cambridge Neuropsychological Test Automated Battery. Six neuropsychological tests on the participants’ motor, learning and memory, attention, and executive functions were administered. Higher consumption of kratom tea (more than three glasses per day or mitragynine levels between 72.5 mg and 74.9 mg) was specifically linked to subpar performance on the paired associates learning task, which reflects deficiencies in visual episodic memory and new learning. Overall, the performance of kratom users was equivalent to that of control participants, and both high (>3 glasses per day) and low (3 glasses per day) use groups performed similarly across all neuropsychological domains. Regular users of kratom did not appear to experience any negative effects from consuming more kratom juice (>3 glasses per day) [36].

Vicknasingam et al. studied the main causes of kratom use, as well as the sociodemographic traits of its users [9]. He included 136 active users in his study, and 76.5% of them had previously used narcotics. He found that the use of kratom had advantages over heroin, including reducing dependence on other drugs, easing opiate withdrawal symptoms, and being more affordable. Many short-term and long-term users claimed to feel more energetic, capable of working hard, and to have a higher sexual desire [9,47]. Another anonymous cross-sectional online survey in the USA was carried out with 8049 kratom users in 2006 [48]. The results of this study showed that middle-aged (31–50 years) and middle-income (over $35,000) people are the main users of kratom, with the main uses being the treatment of pain (68%) and emotional or mental conditions (66%).

As a relatively affordable opiate substitute that does not require a prescription from a doctor, kratom is still readily available for purchase online [49,50]. Since the beginning of the new millennium, kratom-branded goods have been sold in Europe under the names “Kratom acetate” or “mitragynine acetate” [2]. Due to its psychoactive qualities, kratom products have recently been advertised as “incense”, albeit the levels of these active compounds differ depending on the type of kratom utilized, the environment, and the time of harvest. Along with khat and *Salvia divinorum*, kratom was one of the top three plant-based substances according to the United Nations Office of Drugs and Crime’s questionnaire on NPS [51]. Because kratom was infrequently observed in national drug abuse surveys, information on its prevalence is scarce. The 1961 and 1971 conventions do not identify kratom or its active alkaloids, but numerous nations have established regulations for its management, which also cover mitragynine and 7-hydroxymitragynine (7-HMG) [3].

Kratom was one of the most commonly given NPSs, according to internet surveys conducted by the European Monitoring Centre for Drugs and Drug Addiction (EMCDDA) in 2008 and 2011 [4]. It is not currently prohibited in the USA or the majority of Europe. Kratom and mitragynine, as well as 7-HMG, are restricted substances in numerous EU nations, including Denmark, Latvia, Lithuania, Poland, Romania, and Sweden, due to their significant potential for abuse. They are regulated under drug legislation in other nations, including Australia, Malaysia, Myanmar, and Thailand (which legalized the use of kratom and cannabis plants for medicinal use in December 2018). Kratom and mitragynine are regulated in New Zealand by the Medicines Amendment Regulations [4].

## 5. Extraction and Extractive of Kratom

Researchers concurred that solvent type, which also influences the number of plant extracts, dictates the kind of phytochemicals recovered from plant sources. The leaf of kratom, which is extracted with methanol, has been the subject of most research. To obtain alkaloid extract, the methanol extract was produced in acid, followed by alkaline, and chloroform extraction. Using the accelerated solvent extraction (ASE) method on kratom leaves, water, ethanol, and ethyl acetate were employed instead of methanol, the most used extraction solvent because of their lower toxicity, safety, and environmental friendliness [52,53].

Utilizing UHPLC-ESI-QTOF-MS/MS analysis, it was possible to determine how the ASE technique affected the extraction yield, total phenolic content (TPC), total flavonoid content (TFC), and phytochemical profiling of kratom leaf; however, there was no discernible change in the dry yield or mitragynine content following water extraction at various extraction times. The dry yield of the extract and its associated mitragynine content varied between 0.53 and 2.91 g when kratom leaves were extracted using organic solvents of various polarities, such as methanol, ethanol, and ethyl acetate. The ethyl acetate ASE extract of kratom leaf had the greatest TPC (459.78 ± 5.47 GAE mg/g) compared to other ASE extracts when the TPC and TFC for kratom leaf extracts were evaluated. In comparison to other ASE extracts, TFC was noticeably greater in the ASE ethanol kratom leaf extract (194.00 ± 5.00 QE mg/g) [20].

In a different investigation, adding heat and an ultrasonicator to the acetic acid extraction process increased the amount of mitragynine that was extracted. By extending the time, increasing the temperature, and using an ultrasonicator, the % yield of mitragynine increased. The best extract of mitragynine was discovered to be acetic acid, which produced the maximum yield of mitragynine at 2.69 ± 0.12% when heated to 80 °C for 30 min. After being extracted with hexane, dichloromethane, ethyl acetate, ethanol, and 50% acetic acid, purified mitragynine from the kratom extract showed antibacterial action. Extracting using boiling water is another facile extraction method, which uses boiling water followed by dichloromethane partitioning. The three primary indole alkaloids—mitragynine (MG), paynantheine (PAY), and speciogynine (SG)—were isolated by boiling fresh leaves in water and then partitioning them with dichloromethane. The extraction yield was 1.0% (*w*/*w*) under ideal conditions, which was ten times lower than the yield of methanol. The *Stephania venosa* extract produced by this approach was rich in alkaloids. The technique was simple to carry out, affordable, safe for the environment, and quickly scaled up from a laboratory scale to an industrial scale [54].

In some other studies, the conventional approach of extraction was used starting with non-polar (hexane), followed by medium polar (chloroform), and polar (methanol); however, no yield was accounted for in this experiment. The evidence on the use of multiple solvents for extraction and variation in yield content did not the intrigue of why hydroalcoholic extract or Soxhlet apparatus is not yet used for kratom extraction. The consequence of their use in biological function may lead to the creation of a new question.

## 6. Chemistry and Composition-Linked Actions of Kratom

The phytochemical properties of kratom have been extensively reported for many years. Kratom consists of 79 secondary metabolites, a high number of alkaloids, flavonoids, polyphenols, terpenoids, triterpenoids, saponins, and secoirods [16,55,56,57].

**Figure 2:**
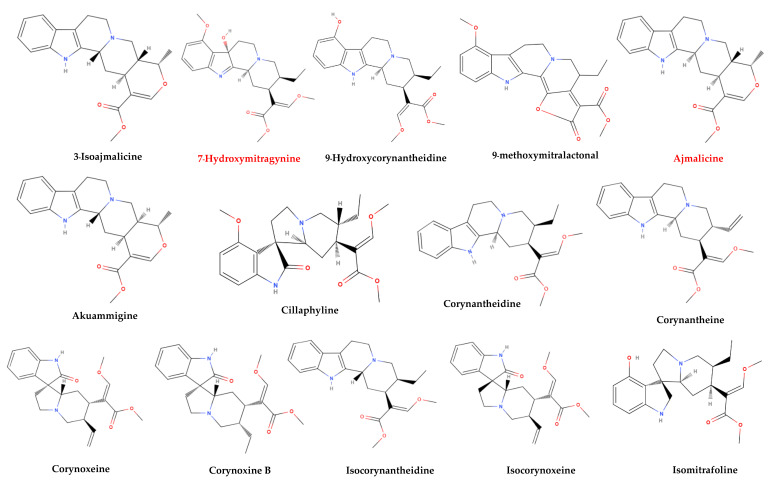

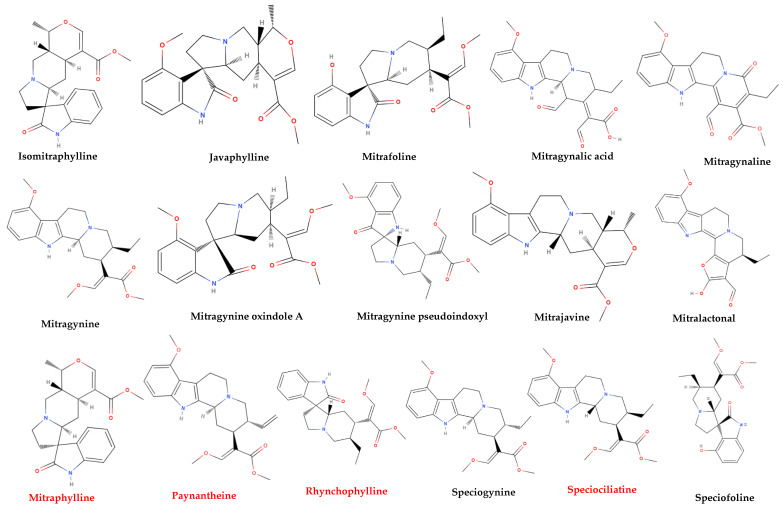
Structural diversity of the chemical compounds from kratom. Red color names signify active compounds with reported pharmaceutical properties.

More than 50 alkaloids from kratom leaves have been isolated during the past 87 years, and six of them were identified pharmacologically active as a psychoactive substance through testing. These six alkaloids are mitragynine (66–67%), paynantheine (PAY)-(8–9%), speciogynine (SG)-(6–7%), 7-hydroxymitragynine (7-HMG)-2%, speciociliatine (SC)-(0.8%), and mitraphylline (<0.1% approximately) (Figure 2) [5,51,58,59]. Other substances that kratom covers likewise have various pharmacological effects: ajmalicine (cerebro-circulant, anti-aggregant, anti-adrenergic, sedative, anti-convulsant, smooth muscle relaxant) [60,61]; akuammigine (stimulant, analgesic, anti-malarial) [62]; ciliaphylline (anti-tussive, analgesic, mild sedative and anti-diarrheal) [63]; corynantheidine (anti-hypertensive, α-1 and α-2 Adrenergic) [64]; corynoxeine (calcium channel blocker, anti-locomotive, anti-Parkinson’s effect) [65]; epicatechin (antioxidant, anti-bacterial, anti-diabetic, anti-inflammatory) [66,67]; 9-Hydroxycorynantheidine (partial opioid agonist) [68]; isomitraphylline (immune-stimulant, anti-leukemic) [69]; isomitrafoline (auxiliary immune-stimulant), isopteropodine (immune-stimulant, anti-bacterial) [70]; isospeciofoline (analgesic, anti-tussive), mitrafoline (anti-hypertensive, anti-amnesic, anti-leukemic), mitraciliatine, mitraphylline (strong muscle relaxant, vasodilator, diuretic), mitraversine, rhynchophylline (calcium channel blocker) [71]; isorhynchophylline, speciofoline, speciophylline (anti-leukemic); stipulatine; tetrahydroalstonine (hypoglycemic, anti-adrenergic) (Figure 2) [10,36,72,73,74,75]. The primary alkaloid in kratom, mitragynine, a corynanthine-like indole alkaloid, was initially isolated by Field in 1921 and has subsequently shown opioid receptor affinity and partial agonist activity. It makes up roughly 1−2% of the dried leaf material [76]. The structure of mitragynine, a white amorphous powder, was first fully determined in 1965 through X-ray crystallography [77] and was found to be soluble in alcohol, chloroform, and acetic acid. Some other indole and oxindole moiety-based major alkaloids possessed by kratom are speciociliatine, speciogynine, paynantheine, and minor alkaloids are isopaynantheine, 7-hydroxy mitragynine (7OH), corynoxine A, corynoxine B, mitraciliatine, corynantheidine [30,37,78,79,80,81,82].

Apart from a high number of alkaloid components, kratom also has some secondary metabolites, various saponins, iridoids, and other monoterpenoids, triterpenoids such as ursolic acid, and oleanic acid, as well as various polyphenols including apigenin, apigenin 7-glycosides, quercitrin, isoquercitrin, rutin, and quercetin type flavonoids. The list of kratom metabolites also includes hyperoside, quercetin-3-galactoside-7-rhamnoside, kaempferol, kaempferol 3-glucoside, epicatechin, caffeic acid, chlorogenic acid, 1-*O*-feruloyl-β-d-glucopyranoside, benzyl-β-d-glucopyranoside, quinovic acid and its derivates, monoterpenes 3-oxo-α-ionyl-*O*-β-d-glucopyranoside, roseoside, secoiridoid, vogeloside, epigeloside, etc. [16,83,84,85,86].

### Nature of Alkaloids from Kratom

Most alkaloids found in kratom are indole alkaloids, including 7-OH, anjmalicine, paynantheine, mitragynine, speciogynine, isopaynantheine, and mitraciliatine. Six other oxindole alkaloids also appear in it: rhynchophylline, isomitrabrylline, isospeciofoleine, speciofoline, and corynoxine A [87]. Speciogynine, paynantheine, isopaynantheine, and speciociliatine are diastereoisomers of mitragynine that are found in these alkaloids [88]. 7-OH is a well-known terpenoid indole alkaloid that descended from mitragynine in 1994. According to Azizi et al. [89], the three Corynanthe alkaloids found in kratom, mitragynine, paynantheine, and speciogynine, all have a distinctive 9-methoxy group that contributes to their biological effect on the central nervous system. Indole alkaloids include akuammigine, ciliaphylline, epicatechin, and isopteropodine (a hetero yohimbine-type oxindole alkaloid), mitraversine (an indole derivative), rhynchophylline (an indole alkaloid), and tetrahydroalstonine (a yohimban alkaloid, an organic heteropentacyclic compound, and a methyl ester). The remaining kratom components are either alkaloids or polyphenolic chemicals. Understanding the nature of these alkaloids is critical for understanding the mechanisms underlying kratom’s purported neuroprotective benefits.

## 7. Toxicology and Toxicokinetics of Kratom

As of yet, nothing is known about the toxicokinetics of kratom in humans, including the metabolic half-life, protein binding characteristics, and elimination rates [90,91]. On the other hand, moderate to high dosages (5 to 15 g) produce opioid-like effects. It has been demonstrated experimentally that low to moderate dosages (1 to 5 g) provide modest stimulant effects to aid employees in overcoming weariness [9]. High dosages (>15 g) are associated with reports of anxiety, irritation, and increased aggression, which have been linked to several unusual consequences [9,38,51]. For some users, the kratom withdrawal effect is quite unpleasant, making it difficult to maintain abstinence, as similar to opioid withdrawal. In a study on animals, Trakulsrichai et al. [92] discovered that the main unfavorable consequences of ingesting kratom “mitragynine tea” were the onset of numbness and an increase in blood pressure and heart rate, which all occurred eight hours after consumption. Using 200 mg/kg of kratom whole alkaloid extract caused rats to die, according to a study [89]. However, 129 kratom users in the USA were found to use 1–3 g of kratom each dose regularly [93]. Of the 129 regular users, 37% consumed kratom as a no optimum dose of kratom is still set a safe dose. A case report of a young kratom user showed that approximately 10–14 days after consumption was stopped kratom metabolites could still be detected in urine. Saturation of enzymatic pathways or high plasma protein binding could account for this situation, but none of this was proven to be right [94]. A significant rise in blood pressure (one hour after administration), acute severe hepatotoxicity, and mild nephrotoxicity were noted in a 14-day intervention of toxicity evaluation using 100, 500, and 1000 mg/kg BW of kratom methanolic extract, even though spontaneous behavior, food, and water consumption, absolute and relative organ weight, and hematological parameters were normal [95]. Kratom was discovered to damage the kidneys and the lungs, resulting in emphysema, over-inflation of the alveoli, and an increase in blood urea and serum creatinine levels [96]. Neither kratom extract nor mitragynine displayed any genotoxicity toward human brain cells in the mouse lymphoma gene mutation assay. The Ames test was used in research, but no mutagenic effects were discovered [97].

Although numerous toxicities and fatal results following the use of mitragynine or kratom have been reported, the fundamental causes are still unknown. Mitragynine is a glycoprotein-P inhibitor, which Rusli et al. [98] demonstrated interacts with significant residues at the nucleotide-binding domain site of glycoprotein-P but not with residues from the substrate binding site (a multidrug transporter for modulating xenobiotic pharmacokinetics mediation of drug–drug interactions). As a result, it is okay to use mitragynine-containing kratom products concurrently with medications that alter the way glycoprotein-P behaves, but not with residues that are involved in substrate binding. As a result, taking mitragynine-containing kratom products at the same time as psychoactive medications that are glycoprotein-P substrates may cause toxicity, possibly having therapeutic implications.

Due to the activation of drug-metabolizing enzymes such as CYP450s and UDP-glucuronosyl transferase, kratom metabolism is primarily hepatic, and there is some evidence that this can alter the metabolism and effectiveness of other medicines (UGT) [99]. Inhibition of CYP3A4, CYP2D6, and CYP2C9 was discovered to be caused by kratom alkaloid extract according to several studies that examined the effects of kratom on human recombinant CYP450 enzyme activities [100]. Mitragynine was substantially degraded in liver microsomes, largely to O-demethylated and mono-oxidated metabolites, according to Kamble et al. [101]. Some other investigations that assessed the effects of kratom on human recombinant CYP450 enzyme activity have confirmed that these enzymes partially account for inter-individual variability in drug metabolism and toxicity due to cytochrome-related genetic variants in humans [100,102]. This finding implies that mitragynine should be administered concurrently with herbal or contemporary medications that follow the same metabolic pathway as herb–drug interactions [103]. Particularly in the case of medications with limited therapeutic windows as carbamazepine, theophylline, digoxin, warfarin, and phenytoin, such combinations may cause severe adverse drug responses [104]. Ulbricht et al. [105] reviews found that kratom is likely to be dangerous if used by individuals with neurologic problems or those who are using neurologic medications like alcohol, sedatives, benzodiazepines, opioids, or goods containing opium, or stimulant drugs like caffeine, caffeine-containing products, cocaine, yohimbine, or related substances. Additionally, it is not recommended to co-administer monoamine oxidase inhibitors (MAOIs). A recent tragic case involving a 27-year-old male who had deadly levels of both mitragynine and quetiapine in his blood was reported [106]. While Yohimbe (*Pausinystalia johimbe*) in combination with kratom has been observed to produce overstimulation and elevated blood pressure, other herbs have also been documented to exhibit drug–drug interactions with kratom [105]. After all, in an inconclusive safety profile of kratom, the overweigh benefits on toxicity and risk can open the window to the use of kratom in respective cases [107].

## 8. Neurological Effects of Kratom

The neurological effects have been defined in the context of the following spectrum of biological events that appeared to be approached by kratom:

The central nervous system actions of kratom’s alkaloids and their derivatives are the subject of growing scientific interest. Two ways that mitragynine’s effects on the nervous system were revealed in 1932 were effects on the autonomic nervous system, which included facilitation of impulse passage affecting both the cranio-sacral and sympathetic divisions, and another effect on the central nervous system, which included excitation of the medulla, likely the motor centers [108]. Kratom was found to show an anti-depressant activity at the behavioral level [27]. Mitragynine, the major compound of kratom, was found to activate the GABAB receptor in a mitragynine-induced conditioned place preference test in rats [109]. Mitragynine was also found to show a weak functional AMPA and NMDA receptor antagonist action [110]. However, the neuroendocrine hypothalamic–pituitary–adrenal axis is overactive, as evidenced by the excess production of monoamine neurotransmitters including serotonin, noradrenaline, and dopamine. Nonetheless, the complex pharmacological profile of raw kratom extracts may be explained by the Mitragyna alkaloids’ apparent diverse activities at other brain receptors, such as adrenergic, serotonergic, and dopaminergic receptors [111]. Chronic mitragynine (5–15 mg/kg; i.p.) injection for 28 days before a working memory test in mice markedly decreased locomotor activity in an open-field test and object identification [28]. Acute oral administration of kratom extract had no discernible effects on mice’s short-term memory or their ability to coordinate their movements when tested with the rota-rod and the Y-maze, but it did increase their exploratory activity in the Y-maze [112]. A human study published in 2018 found that frequent kratom users’ motor, memory, attention, and executive function were unaffected by consuming more than three glasses of kratom juice per day [36]. Chronic morphine, Δ-9-tetrahydrocannabinol, or kratom administration impaired spatial learning and memory processing [113]. The methanolic extract of kratom (100–1000 mg/kg) was reported to promote learning by demonstrating the latency as a deficiency in memory consolidation of a passive avoidance test. In a two-way active avoidance task, the methanolic extract of kratom had no discernible effects on long-term memory consolidation. The methanolic extract inhibited long-term potentiation (LTP) induction but promoted short-term potentiation in hippocampal field excitatory postsynaptic potentials (fEPSP), demonstrating the impact of extract constituents on the brain’s learning and memory pathways [114].

### 8.1. Kratom, an Indole-like Alkaloid for Neurological Effects

**Figure 3:**
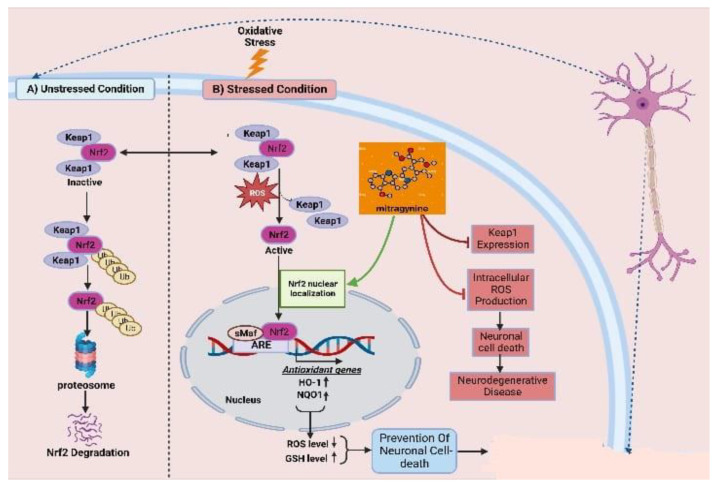
Keap1–Nrf2-linked neuroprotective effects of kratoms’ major compound mitragynine. Oxidative stress leads to dissociation of the Keap1–Nrf2 conjugation, homeostatic condition, and releases the Nrf2. The resulting Nrf2 becomes translocated to the nucleus and upregulates the expression of antioxidative genes. Mitragynine is proposed to facilitate the translocation of Nrf2 to the nucleus, while Keap1 expression seems responsible for neuronal cell death and other neurodegenerative diseases.

As one of the most abused plant psychotic drug sources, kratom possesses a powerful psychoactive compound in the form of mitragynine, which demonstrates opioid-like behavioral effects and results in neuroplasticity in the reward system of the brain. There are evident and reported cognitive impairments associated with its chronic administration. By combining increased efficacy with better tolerability and a sparing of opioids, multimodal analgesic strategies are paving the way for major improvements in the management of pain. The association of analgesics with different mechanisms of action has proven to be a successful strategy for the treatment of a wide range of pain conditions, minimizing side effects and maximizing the advantage of additive and synergistic effects of the individual agents. Mitragynine, the most common and concentrated indole alkaloid of kratom, is postulated to be involved in the regulation of the Keap-1/Nrf-2 pathway to ensure neuroprotection. The mechanism by which mitragynine exerts its complex effects adrenergic, serotonergic, and opioid-like activity is structurally and pharmacologically distinct from that of traditional opioids. In addition to mitragynine and many of this category’s alkaloids, many of them may play a pivotal role in regulating the overproduction of intracellular ROS, which contribute to neuronal cell death via H_2_O_2_ exposure. It has been shown that the activation of antioxidative genes of Nrf2, such as HO-1 and NQO1, is primarily dependent upon the nuclear translocation of Nrf2 (Figure 3) [115]. This implies that Nrf2 translocation from the cell cytosol to the nucleus plays an important role, while indole-like alkaloids (e.g., prenylated alkaloids) inhibit Keap1, resulting in Nrf2 nuclear translocation. By activating Nrf2, HO-1 and NQO1 are expressed, resulting in a decreased level of ROS and an increased level of GSH, thereby protecting neurons from oxidative damage.

### 8.2. Anti-Inflammatory Effects Leading to Neuroprotective Effects

**Figure 4:**
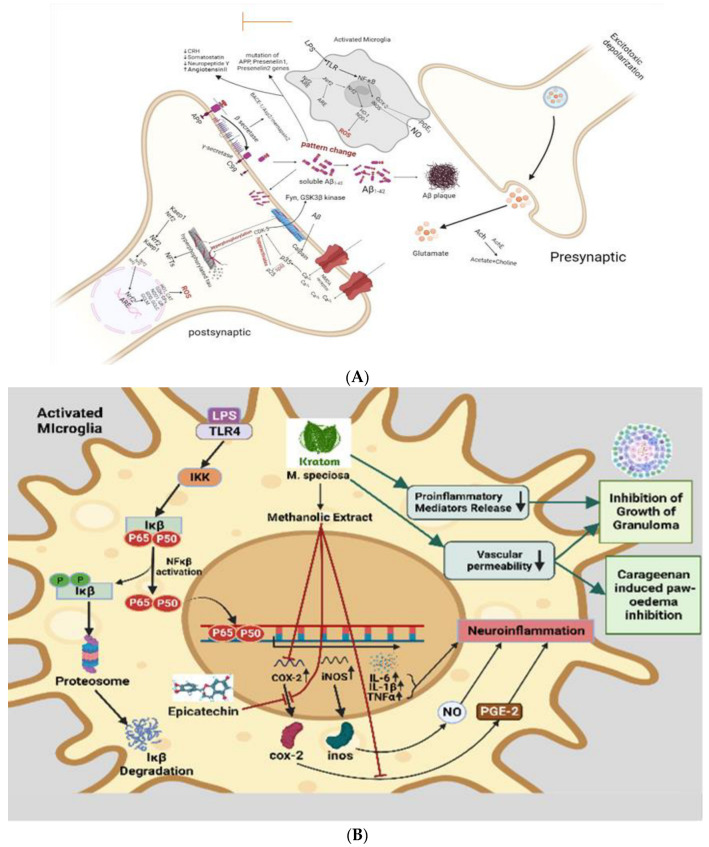
(**A**) Speculated neuroprotective mechanism of kratom showed that inhibit COX-2 activity. In the neuroinflammation pathway, microglial cells are activated by LPS. As a result of oxidative stress, and inflammatory cytokines, some proteins are phosphorylated and activated and result in the phosphorylation and ubiquitination of NF-κB. Then, NF-κB is secreted to the nucleus where it can bind to a specific binding site to activate the transcription and translation of inflammatory cytokines (TNF-α and IL1-β) and proteins (COX-1, COX-2, and iNOS), which are released from the microglia. Kratom inhibits COX-2 activity. (**B**) Anti-inflammatory action-based neuroprotective effect of kratom. Inflammatory status in activated microglia is initiated through LPS-mediated TLR, IKK, Ikβ, and NFkβ activation leading to the synthesis of interleukin and TNFα which are thought to be neuroinflammatory modulators. While kratom and its alkaloids are postulated to inhibit COX-2, proinflammatory mediators and a decrease vascular permeability imply the contribution of kratom in neuroprotection.

In a recent study, kratom has been shown to inhibit proinflammatory mediators release, reduce vascular permeability, and enhance immunity [107]. Activated inflammatory cells at the site of infection release inflammatory mediators like cytokines, arachidonic acid, and chemokines, which in turn trigger signal transduction cascades and changes in transcription factors like nuclear factor kappa B (NF-κB), signal transducer and activator of transcription 3, activator protein-1, NF-E2-related factor-2, nuclear factor of activated T cells, and hypoxia-inducible factor-1*α* (HIF1-*α*). initiation of cyclooxygenase-2 (COX-2) (Figure 4A), inducibility of nitric oxide synthase (iNOS), and high expression of inflammatory cytokines such as tumor necrosis factor-α (TNF-α), interleukin-1β (IL-1β), IL-6, and chemokines (CXC chemokine receptor 4) [116,117]. Epicatechin has effective anti-inflammatory effects. One of the most potent inflammatory mediators is PGE2. Prostaglandin PGE2 is produced by COX-1 and COX-2, which are cyclooxygenases involved in the inflammatory pathway. Previous research demonstrates that the methanolic extract of kratom inhibits COX-2 mRNA and protein expression in RAW264.7 macrophage cells, as well as PGE2 production [118]. The Aβ plaque comprises Aβ peptides derived from APP through enzymatic cleavage via (α, β, and γ) secretases. Aβ1-42 readily aggregates and forms the plaque that activates calpain and deregulates p35 into p25, which hyperactivates CDK5 and leads to hyperphosphorylation of tau and the formation of NFTs. β amyloid plaques promote neurotoxicity or activation of microglia by upregulating NF-κB and AP-1 transcription factors, which in turn release ROS and pro-inflammatory cytokines like NO, PGE2, IL-1, IL-6, COX-2, and TNF-α that damage cholinergic neuron (Figure 4B). These pro-inflammatory cytokines also directly stimulate astrocytes, which create their cytokines to increase inflammatory signals, leading to neuroinflammation and neurodegeneration. Daily intraperitoneal injection of 100–200 mg/kg kratom methanolic extract also had a significant inhibitory effect on the development of granuloma tissue, as demonstrated by the proliferation of modified macrophages, fibroblasts, and highly vascularized, reddish mass tissue, as well as a significant inhibitory effect on the progression of carrageenan-induced paw oedema. In spite of some question marks, inflammation response mechanism, inflammatory cytokines, activation of the inflammasome, and metabolic syndrome as a cause of inflammation in neurotoxicity show that anti-inflammation and neuroprotection are strongly correlated [119]. In the study, the authors suggested that kratom’s anti-inflammatory properties could be attributed to its inhibition of the release of proinflammatory mediators and its effect on vascular permeability, as well as enhanced immunity and stimulation of tissue repair and healing processes [21].

### 8.3. Analgesic and Anti-Nociceptive Effects

The first case of using kratom was as an anesthetic by a patient with chronic pain [120]. In both a concentration-dependent and a time-dependent manner, indole alkaloids such as mitragynine and other derivatives (7-HMG, SC, PAY, and SG) isolated from kratom inhibit electrically induced contractions. Naloxone reversed the opioid receptor agonistic action of electrical stimulation on the guinea pig ileum using the switch contraction of the ileum, which was measured by the opioid receptor agonist action of electrical stimulation [78,121,122]. There was a delay in nociceptive responses to noxious stimulation by both methanolic and alkaloid kratom leave extracts in mice in the hot-plate test, but not in the tail-flick test [123]. Furthermore, a methanolic extract of kratom was shown to possess antinociceptive activity when it significantly reduced writhing responses and pain sensations in a study where acetic acid was used as the writhing stimulus and the formalin test was administered [21]. Comparing the antinociceptive effects of various oral kratom extracts with morphine in rats, researchers concluded that alkaloids (20 mg/kg), methanolic extracts (200 mg/kg), and aqueous extracts (100–400 mg/kg) all prolonged the latency of nociceptive responses in both the hot plate and the tail flick-tests. Administrating naloxone before the administration of morphine blocks its effects, suggesting that the opioid receptor may play a partial role in mediating those effects [124]. In comparison to 5 mg/kg morphine, these effects were less pronounced, but they were more evident than after 100 mg/kg paracetamol [125]. The anti-nociceptive activity of the alkaloid extract of kratom was potentiated by co-administration of caffeine (25 mg/kg, p.o.) and codeine (3 mg/kg, p.o.) in a hot plate test in rats [90,126]. 7-HMG exhibits more potent antinociceptive activity in the tail-flick and hot-plate tests than morphine when administered subcutaneously or orally. The ability of 7-HMG to penetrate the blood–brain barrier (BBB) and exert a more rapid effect than morphine has been attributed to its higher potency and rapid effect [68,122]. 7-HMG was also confirmed to have a high level of potency in opioid receptors. The analgesic properties of mitragynine are 13 times greater than those of 7-HMG, while 3–4 times higher for mitragynine [23,127]. There was also evidence that 7-HMG, a minor constituent of kratom, was 46 times more potent as an analgesic than mitragynine in another study [122]. In humans, it exerts sedative and analgesic effects at higher doses and stimulant effects at low doses [38]. Due to this characteristic of the plant, drug addicts have been highly tempted to abuse it [128]. In the tail-flick test in mice, intracerebroventricular administration of mitragynine and mitragynine pseudoindoxyl had an antinociceptive effect with an ED_50_ estimate of 60.22 nM and 6.51 nM, respectively. The antinociceptive effects of mitragynine and mitragynine pseudoindoxyl were blocked by naloxone, indicating that they are mediated by opioid receptors [78,121]. By blocking 1-opioid receptors, the antinociceptive effect of 7-HMG was eliminated in both tail-flick and hot-plate tests since its antinociceptive action is dose-dependent and predominantly mediated through these receptors [129]. It has been shown that mitragynine binds strongly to the μ-opioid receptors and has analgesic, respiratory depression, and euphoric effects [130,131]. A part of the antinociceptive activity of 7-HMG has also been attributed to the supraspinal-μ and δ-opioid receptors [6,130,131]. In addition to alleviating withdrawal symptoms, kratom can be used to diminish the effects of opium addiction. However, it has a lower affinity for the κ-receptor [131]. Through presynaptic dopamine actions, the κ-receptor exhibited analgesic and depressive effects on locomotor activity [132]. 7-HMG’s supportive actions are partially mediated by μ and δ-opiate receptors [133]. In contrast, a study revealed that kratom powder has less affinity for the μ-opioid receptor than morphine [134]. In mice, the head-twitch reaction brought on by activating postsynaptic 2-adrenoceptors can be reduced by mitragynine and the 5-HT2A receptor antagonist ritanserine. Mitragynine and 7-hydroxymitragynine may produce antinociceptive synergism with adrenergic-α2 (Aα2R) and μ-opioid receptor agonists, according to Obeng, S [135]. Mitragynine may potentially generate hypothermic synergism when paired with Aα2R agonists. The improvement in positive and negative psychotic symptoms by the methanolic extract of kratom may be attributable to the inhibition of D_2_ and 5-HT_2_ receptors [23,136]. Mitragynine may inhibit NG108-15 cell adenylyl cyclase via opioid receptors. Mitragynine can limit neurotransmitter release by reversibly inhibiting neuronal Ca^2+^ channels, which may lead to a reduction in neurotransmitters and an inhibition of pain transduction [122]. In the heart, kratom inhibits hERG-mediated K+ currents and prolongs action duration, constituting a major risk of cardiotoxicity due to blockage of the human Ether-a-go-go-Related Gene (hERG) channel [137].

### 8.4. Neurological Effects by Gene Regulation

**Figure 5:**
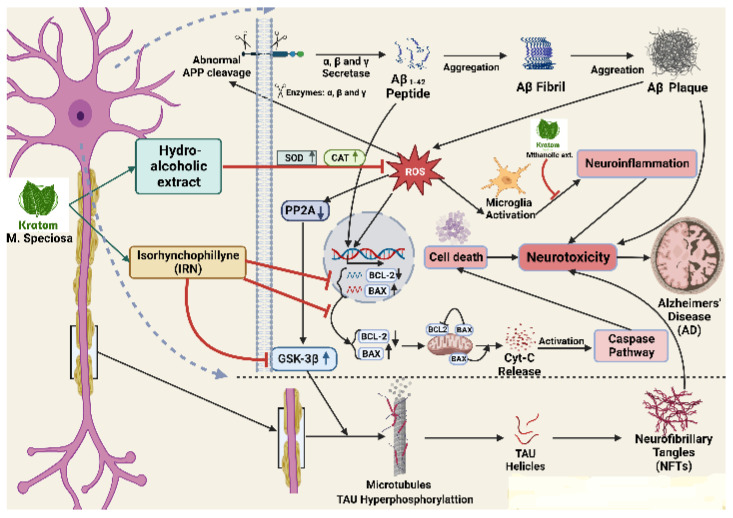
Cascade of gene regulation by kratom to control neuroinflammation. Abnormal APP cleavage eventually assists the formation of Aβ plaque which is stimulated by ROS and increases the neuroinflammation in many ways including BCL-2 decrease, BAX increase, microglia activation, and PP2A activation leading to GSK-3B activation. The activation of GSK-3B finally increases NFTS formation. Kratom’s alcoholic extract inhibits ROS production and its products (IRN) inhibit GSK-3B to substantially inhibit the NFTs formation.

Figure 5 has been summarized to show the gene cascade for neuroprotection by kratom. An interesting finding has been those two alkaloids, bufanidrine (2) and buphanisine (3), have a high affinity for the serotonin reuptake transport protein (SERT). The neuroprotective effects of the Amaryllidaceae alkaloids were considered to be associated with the 1,3-dioxole moiety in those alkaloids, which was thought to be responsible for their neuroprotective effects in AD [138,139,140]. According to previous studies on MAO inhibitory effects of some plant alkaloids, bitter leaf alkaloid-rich extract (BLAE) might produce these effects as a result of its constituent alkaloids [141,142]. Several amine neurotransmitters, such as noradrenaline, dopamine, and serotonin, are oxidized by the MAO enzyme, which is a strategic neuronal enzyme [141]. Furthermore, elevated MAO activity has been directly linked to AD and Parkinson’s disease (PD) because of excessive enzymatic depletion of neuroactive amines and the generation of free radicals which initiate and propagate oxidative stress in AD brains [141,143,144,145]. As a result, inhibiting MAO activity is a valuable restorative methodology for AD and PD [146].

As a potential treatment for AD, it has been reported that repression of acetylcholine esterase (AChE), butylcholine esterase (BChE), ATPase, ADPase, and MAO activity may be effective in combination. The enzymes superoxide dismutase (SOD) and catalase (CAT) are also important antioxidant enzymes that serve to prevent the toxicity of hydrogen peroxide (H_2_O_2_). They were observed to be markedly increased in the brain tissue with the treatment of kratom hydroalcoholic extract in an animal model [147]. It has been discovered that elevated amounts of Aβ oligomers promote the development of oxidative stress, neuroinflammation, synapse loss, and nerve cell death. In another current study, mitragynine was shown to inhibit the enzyme acetylcholinesterase (AChE) involved in AD [148]. α-synuclein expression was established to increase in PD and several alkaloids, such as physostigmine, that are reported to attenuate the expression of the α-synuclein gene. ROS, which is also the primary cause of the aberrant aggregation of Aβ peptides that causes the progression of AD which is particularly sensitive in the brain [149]. The alkaloid Isorhynchophylline is beneficial for treating AD because of its neuroprotective properties through lowering the levels of Bcl-2/Bax gene expression by reducing Aβ-tempted neuronal apoptosis of neurons in the hippocampus. In the Aβ-transgenic CL2006 and CL4176 strains, palmatine, a naturally occurring isoquinoline alkaloid, greatly reduced Aβ-induced paralysis and showed neuroprotective benefits. A variety of antioxidant defense mechanisms, which include the involvement of antioxidant enzymes, like SOD and CAT, mediate the removal of excessive ROS. In wild-type nematodes, palmatine increased the expression of heat shock genes (*shsp)*, such as *hsp*-16.11, *hsp*-16.2, and *hsp*-16.49, and it increased the intensity of *hsp*-16.2p: GFP fluorescence in transgenic CL2070 nematodes. As a result, it is probable that sHSP’s enhanced expression reduced protein aggregation and reduced Aβ toxicity, indicating that sHSP is crucial to Palmatine’s neuroprotective benefits. Heat shock factor (HSF-1) is a transcription factor that is known to have a major role in regulating the production of sHSP. Its decreasing activity has been linked to several detrimental processes that occur in neurodegenerative diseases [150,151]. The results are from previous studies that suggest HSF-1 is involved in the inhibition of Aβ toxicity [151,152]. As a result, the regulator HSF-1, and modulation of the expression of its target genes, including *hsp*-16.11, *hsp*-16.2, and *hsp*-16.49, are involved in palmatine-mediated suppression of Aβ toxicity. In comparison to morphine, speciociliatine, and mitragynine had DNA protection capacities that were, respectively 1200- and 20- fold higher. In a dose-dependent manner, mitragynine, the main component of the alkaloid extracted from kratom, was administered at concentrations ranging from 0.5 to 20 g/mL. This resulted in a significant inhibition of the mRNA expression of COX-2 induced by LPS, which was followed by a decrease in PGE 2 production, implying mitragynine’s anti-inflammatory effects [118]. The contribution of all these alkaloids is hypothesized to impact the neuroprotective effect of alkaloids of alkaloid-rich plant products kratom either in a direct or in a cascade mechanism of neuroprotection. 

### 8.5. Antioxidative Effects

**Figure 6:**
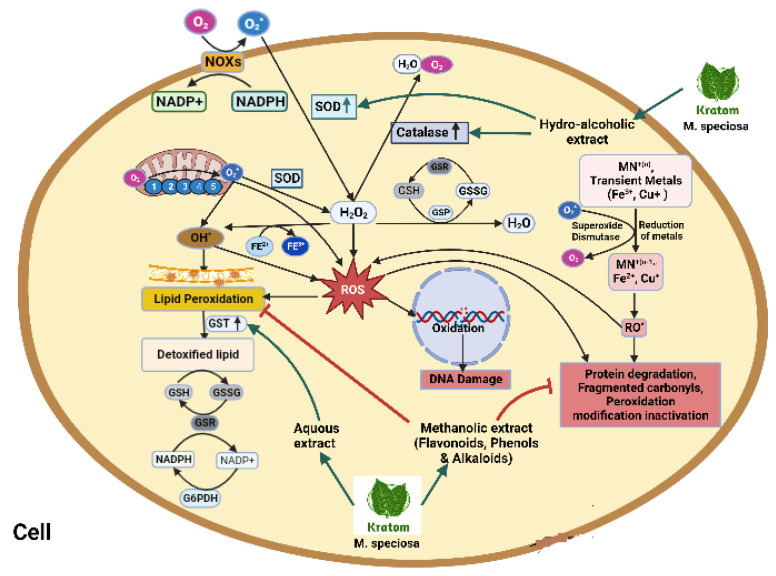
Activation of NADPH oxidase, dual oxidase, and nitric oxidase subsequently reduces SOD-, CAT-, and GPX-producing huge free radicals in a biological system. Hydrogen peroxide produces ROS which ultimately activates lipid peroxidation, protein degradation, DNA damage, and fragmented carbohydrate. Kratom upregulates SOD, CAT, and GST and detoxifies lipids through an oxidoreductase cascade. Kratom and its hydroalcoholic extract inhibit lipid peroxidation and inhibit protein degradation and fragmented carbohydrate.

By limiting the start or growth of oxidative chain reactions, antioxidants are those substances that can delay or hinder the oxidation of lipid or auxiliary molecules [153]. They can extend shelf life and neutralize free radicals by postponing the oxidation of lipids. Antioxidants are available in both natural and synthetic forms. While synthetic antioxidants are produced using synthetic chemicals, natural antioxidants are obtained by extracting natural substances that can snare free radicals [154]. However, numerous types of research on the potential of naturally occurring antioxidants produced from plants have been conducted due to worries about the adverse effects of utilizing synthetic antioxidants. These can prevent degenerative illnesses and food-based fat oxidation [155]. Kratom exhibits antioxidant action, which has primarily been attributed to the presence of polyphenolic substances such as flavonoids and alkaloids (Table 1). DPPH free radical scavenging, lipid protection of DNA, and prevention of metal-induced protein oxidation against H_2_O_2_-induced oxidative stress were all used to assess antioxidant capabilities [156]. According to reports, the polar nature of the antioxidant biomolecules may be seen in the fact that aqueous and methanol extracts from several of plant sources have better free radical scavenging action than dichloromethane or ethyl acetate extracts [154]. By using several techniques, including the 2,2-diphenyl-1-picrylhydrazyl (DPPH), reducing power, oxygen radical absorption capacity test (ORAC), FRAP, and CUPRAC procedures, the relevance of antioxidant activity in kratom was revealed [157]. Additionally, 100 mg/kg of the kratom aqueous extract significantly increased the specific activity of glutathione-S transferase (GSTs) by 129% compared to the control [89]. Another study showed the ethanolic extract of kratom exposed antioxidant effects on DPPH, and phytochemical screening [16,18]. Kratom was already reported to inhibit the release of proinflammatory mediators, especially NF-κB, interleukins, and cytokines. And antioxidants exert a regulatory effect on the expression of pro-inflammatory cytokines [158]. The comprehensive pathways and links associated with the antioxidative potential of kratom are presented in Figure 6.

## 9. Other Pharmacological Effects

The preceding pharmacological effects of kratom for controlling the disease with several animal and human case studies are shown in Table 2.

### 9.1. Antibacterial Effects

Antioxidants are compounds that resist ROS and free radicals to prevent carcinogenesis, cardiovascular, and aging [156]. Kratom’s antibacterial properties may protect the brain from bacterial infection and bacterial components such as LPS. LPS, a component present in the outer membrane of some bacteria, has the potential to cause an inflammatory reaction in the body. The core saccharides in all Gram-negative bacteria with LPS are identical and mostly composed of N-acetylglucosamine, glucose, galactose, heptose, phosphate, and ethanolamine. Kratom can also influence the immune system’s response to bacterial infections. LPS’s inflammatory action is caused by the lipid A [159]. LPS causes the production of a wide range of inflammatory cytokines from human peripheral mononuclear blood cells, including IL-1, IL-6, IL-8, and TNF-α on endothelial cells [160]. Excessive or uncontrolled inflammation can harm the brain. The processes through which LPS activates cells and triggers inflammatory responses have received a great deal of attention. The methanolic extract of kratom shows a high content of phenolic and flavonoid compounds, and the result of DPPH and microbial testing against *Salmonella typhi* and *Bacillus subtilis* displayed antioxidant and antibacterial effects respectively [16], while *Salmonella typhi* outbreak is historically reported to cause Malawi–Mozambique disease in 2009 [161]. According to the study, epicatechin (kratom’s phytoconstituent) acts against the bacteria *H. pylori* which is responsible for the progression of neurological disorders [162]. At a dose of 100 mg/kg, kratom aqueous extract significantly increased glutathione-S-transferase (GSTs) specific activity at various levels, demonstrating its antibacterial actions to treat intestinal infections.

### 9.2. Gastrointestinal Effects

The bidirectional gut–brain axis connects the gastrointestinal system and the brain, facilitating communication between the stomach and the central nervous system and impacting different physiological processes, moods, and behavior. This connection encompasses neuronal pathways, neurotransmitters, hormones, immunological responses, and the gut microbiome, emphasizing the significance of gut health for general brain function and mental well-being [163]. Kratom extract-treated rats showed immediate and long-term effects of lower food and water intake, as well as less tendency to acquire weight [22,164]. By reducing defecation frequency, the overall diarrheal score, intestinal transit (by a single dosage), and fecal weight in rats with castor oil-induced diarrhea, methanolic kratom extract demonstrated anti-diarrheal benefits. However, because pretreatment with naloxone did not affect the frequency of feces, repeated treatments with this extract did not result in any appreciable alteration in the intestinal transit and fluid. Excitatory and inhibitory impulses from the enteric nervous system are the primary mechanisms by which the small intestine’s gastrointestinal motility is regulated. Since parasympathetic and sympathetic fibers directly link the central nervous system with the digestive tract and the local neural system with gastrointestinal hormones, kratom extract may have an impact on pathways other than opioid receptors [165]. Mitragynine administration into the fourth ventricle of anesthetized rats caused a dose-dependent inhibition of 2-deoxy-d-glucose-stimulated gastric acid secretion, though its effects were reversed by naloxone, indicating the involvement of opioid receptors. Mitragynine administration centrally did not affect the basal gastric acid secretion into the lateral ventricle. In addition to having an impact on anorexia and weight loss, mitragynine also has a direct inhibitory effect on neurons in the lateral hypothalamus [166]. In addition, subcutaneous administration of 7-HMG to mice inhibited their gastrointestinal transit [68]. Ciliaphylline is a minor alkaloid and showed anti-diarrheal effects.

## 10. Adverse Effects/Abuse of Kratom

Studies on animals and humans have demonstrated the toxic properties of kratom preparations. A total of 428 cases of Kratom use were recorded from 2011 to 2015, according to the Centers for Disease Control and Prevention, USA [167]. According to The Food and Drug Administration, 44 people died from Kratom use in 2018, with mitragynine being a contributing factor [168]. Kratom withdrawal symptoms and side effects are described in some case reports shown in Table 3 [169]. Adverse effects of kratom include nausea, vomiting, tremor, diaphoresis, tachycardia, hypertension, hypothyroidism, elevated creatinine phosphokinase concentrations, dry mouth, headaches, intrahepatic cholestasis, dizziness, itching, fatigue, weight loss, sweating, and heart palpitation [6,46,170,171]. Due to these findings, 15 businesses that were illegally selling kratom were issued warning letters by the institution. Because in many cases other products are consumed along with Kratom, it is not always clear which substances can produce these effects.

**Table 2:** Other biological and pharmacological effects of kratom.

Treatment with Doses	Nature of Kratom Product	Experimental Model	Major Findings (Molecular Changes)	Reference
Anti-bacterial				
Kratom	Methanolic extract (3.12 to 6.25 mg/mL)	*Salmonella typhi* and *Bacillus subtilis*	Minimum inhibitory concentrations (MICs) by the broth dilution method	(Parthasarathy, Bin Azizi et al., 2009) [16]
	Mitragynine (40 mg/kg), alkaloid extract (100 mg/kg)	Adult male Wistar rats	Detoxification and elimination of permethrin	(Srichana, Janchawee et al., 2015) [19]
Gastrointestinal effects				
Kratom	Methanolic extract (50, 100, 200 and 400 mg/kg)	Adult Wistar rats	Protection against castor oil-induced diarrhea, ↓ intestinal transit	(Chittrakarn, Sawangjaroen et al., 2008) [165]
	mitragynine (3–30 μg)	Male Wistar rats	↓ 2-deoxy-d-glucose-stimulated gastric acid secretion	(Tsuchiya, Miyashita et al., 2002) [166]
	7-hydroxymintragynine (ED50 = 1.19 mg/kg)	Male ddY-strain mice	↓ Gastrointestinal transit and significantly antagonized by β-funaltrexamine hydrochloride (β-FNA) pretreatment, but slightly antagonized by naloxonazine	(Matsumoto, Hatori et al., 2006) [68]
Muscle relaxant				
Kratom	Methanolic extract (10–40 mg/mL), mitragynine (2 mg/mL)	Wistar rats	Blockade of nerve conduction, amplitude, and duration	(Chittrakarn, Keawpradub et al., 2010) [172]
	Potential to inhibit enzyme activity			
Kratom	Methanolic extract	Three main CYP450 enzymes: CYP2C9, CYP2D6, and CYP3A4	Most potent effect on CYP2D6 at IC50 (3.6 ± 0.1 μg/mL)	(Hanapi 2010) [173]
	Alkaloid extract	CYP450 enzymes, Quinidine (CYP2D6), ketoconazole (CYP3A4), tranylcypromine (CYP2C19), and furafylline (CYP1A2)	Most potent inhibitory effect on CYP3A4 and CYP2D6 at IC_50_ values of 0.78 µg/mL and 0.636 µg/mL	(Kong, Chik et al., 2011) [100]
Anti-diabetic				
Kratom	Water extract 0.6 mg mL^−1^	L8 muscle cells	↑ Glucose transporters (GLUT1)	(Purintrapiban, Keawpradub et al., 2011) [174]
Anti-hypertensive				
Kratom	Methanolic extract (100, 500, and 1000 mg/kg)	Male Albino rats	Blood pressure (diastolic: 102.7 ± 0.72, 98.74 ± 7.95 and 86.85 ± 3.34), and ↑ ALT, AST, albumin, triglycerides, cholesterol, albumin levels	(Harizal, Mansor et al., 2010) [95]
Weight reduction				
Kratom	Mitragynine (45 and 50 mg/kg)	Male Wistar rats	↓ Food and water intakes	(Kumarnsit, Keawpradub et al., 2006) [22]
	Mitragynine (100 mg/kg)	Male and female Sprague-Dawley rats	↓ Food intake, ↓ Body weight of female rats, and ↑ liver weight of both male and female rats	(Sabetghadam, Ramanathan et al., 2013) [164]

**Note:** Upper-directed arrows indicate upregulation and lower-directed ones indicate downregulation.

**Table 3:** Cases reported for adverse effects of kratom.

Uses Pattern	Side Effects of Kratom	Condition	History	Reference
For 1 month, kratom leaf tea is brewed with Datura stramonium	4–5 mm pupils, minimally reactive, roving conjugate gaze, and spasticity of lower extremities with manipulation	Chronic pain after post-colostomy surgery	64 years male	(Nelsen, Lapoint et al., 2010) [175]
Powder of leaf 4.6–7 to 8.6–14 g/day for 2 weeks	Loss of appetite, fever and chills, slight abdominal discomfort, concomitant brown discoloration of the urine, jaundice, and pruritus	Intrahepatic cholestasis	25 years male	(Kapp, Maurer et al., 2011) [176]
Kratom tea 4 times a day for 3.5 years	A generalized tonic-clonic seizure lasting 5 min, pulse 123 beats per min	Tonic colonic seizure	43 years male	(Boyer, Babu et al., 2008) [128]
1 tablespoon of powder daily for 3 months	Jaundice, dark urine, mild confusion, and liver injury	Cholestatic hepatitis	58 years male	(Dorman, Wong et al., 2015) [177]
6 g Kratom capsules daily for 2 weeks	Palpation of the right upper quadrant (RUQ) in the presence of vomiting, fatigue, abdominal pain, and brown urine	Hepatomegaly	21 years male	(Griffiths, Gandhi et al., 2018) [170]
Sixty tablets over 1 week	A yellowish appearance to the skin, usually associated with nausea, fatigue, joint pains, night sweats, pale stools, and dark urine	Hepatitis	32 years male	(Tayabali, Bolzon et al., 2018) [171]
Herbal drug Kratom	Distention, mass, tenderness, rebound, sternal pleuritic chest pain, mild shortness of breath, mild cough, mild coughing, and mild chest pain	Intrahepatic cholestasis	38 years male	(Riverso, Chang et al., 2018) [178]
A tablespoon of crushed leaves (−1.5 g/d)	Yellow discoloration of eyes and skin, mild fatigue, jaundice	Intrahepatic cholestasis	52 years male	(Fernandes, Iqbal et al., 2019) [179]
Green-colored herbal powder supplement for a few weeks with increasing daily dosage	Pupils were pinpoint and not reactive to light and cool peripheries, the abdomen and pelvis revealed cholestasis without cholecystitis	Intrahepatic cholestasis	36 years male	(Palasamudram Shekar, Rojas et al., 2019) [180]
Kratom tea for 2 weeks	Tea-colored urine, malaise, fatigue, and intermittent subjective fever	Acute hepatitis	31 years male	(Mousa, Sephien et al., 2018) [181]
Kratom capsules for 3 weeks	Dark urine, pruritus, subjective fevers, fatigue, nonbloody, nonbilious emesis, nonicteric sclera, and sublingual jaundice	Hepatitis	47 years male	(Osborne, Overstreet et al., 2019) [182]

## 11. Critical Remarks and Insights

Why kratom, the abusive plant, has been turned into one of the widely used plant sources is partially answered through its undeniable window of valuable compounds. How narcotics become the proposal for neuroprotection has been figured out through the definitive and coordinated mode of neurodegeneration. Whether and to what extent the toxicity of kratom could matter for future drug discovery is inclusively summated in this review. Why the alkaloids, not all but some of them, due to their nature, target selective genes to help neuroprotection is answered to some extent for the future direction of using alkaloids in neuronal abnormality. Nonetheless, the unraveled potential of mitragynine and 7-OH mitragynine might be the pivotal issue to be explored for the best use of kratom based on the interactions summarized in the Table 4. Research on the interaction between neuroprotection-related genes and mitragynine as well as 7-OH needs to be unfolded through a network-pharmacological assessment. Molecular dynamics simulation could be another spotlight proposal to evaluate the biological stability of their interaction for future drug discovery. 

## 12. Conclusions and Future Prospective

In spite of both unsafe and beneficial effects of kratom, a very recent report on kratom as neuroprotective, as well as other neuro-supportive benefits including antioxidant and anti-inflammatory potential, will undoubtedly be allured for its neuroprotective effects. However, all the isolated compounds, except mitragynine and 7-OH mitragynine, are yet to be studied for their biological activities to conclude an unambiguous use of kratom as neuroprotective. Further dose-response preclinical and clinical studies are needed to ensure the neuroprotective effects of kratom affirming its advanced toxicity study. 

## Figures and Tables

**Table 1:** Comprehensive pre-clinical studies for the physiological effects of kratom. Upper-directed arrows indicate upregulation and lower-directed ones indicate downregulation.

Antioxidative Effect				
Treatment with Doses	Nature of Kratom Product	Experimental Model	Major Findings (Molecular Changes)	Reference
**Kratom**	Methanolic, water, alkaloids	In vitro	The high content of phenolic, flavonoid compounds and the result of DPPH, high antioxidant activity in methanolic extract	(Parthasarathy, Bin Azizi et al., 2009) [16]
	Ethanolic extract	In vitro (DPPH)	The IC_50_ value of 38.56 μg/mL	(Yuniarti, Nadia et al., 2020) [18]
	Aqueous extract (100 mg/kg)	Male Sprague Dawley rats	↑ Glutathione transferase (GSTs) activity	(Azizi, Ismail et al., 2010) [89]
**Neurophysiological**				
**Kratom**	Mitragynine (5, 10 and 15 mg/kg)	Male ICR mice	Mitragynine neither altered locomotor activity nor its high or low dose	(Apryani, Hidayat et al., 2010) [28]
	Methanolic extract (0.008%)	Male Sprague Dawley rats	↓ Field excitatory post-synaptic potentials (fEPSP) in the CA1 region concentration-dependently, and blocked long-term potentiation (LTP)	(Senik, Mansor et al., 2012) [114]
**Anti** **-inflammatory**				
**Kratom**	Methanolic extract (100–200 mg/kg)	Male Sprague Dawley rats	Dose-dependently suppressed the development of carrageenan-induced rat paw edema, and ↓ granulomatous tissue formation at 200 mg/kg	(Mossadeq, Sulaiman et al., 2009) [21]
	Methanolic extract (10 and 20 g/mL)	RAW264.7 macrophage cells	↓ mRNA expression of COX-2, ↓ PGE2 production, and ↓ COX-1 expression	(Utar, Majid et al., 2011) [118]
**Analgesic** **/Anti** **-nociceptive**				
**Kratom**	7-hydroxymitragynine (ED_50_ = 0.80 mg/kg, and ED_50_ = 0.93 mg/kg)	Male ddY-strain mice and male albino guinea pigs	4.4–5.7 times more potent as μ-opioid agonist than morphine in tail-flick and hot-plate test	(Matsumoto, Hatori et al., 2006) [68]
	7-hydroxymitragynine (100 nM), speciociliatine (30 μM)	Male Albino Dunkin–Hartley guinea pigs	↓ Twitch contraction and 7-hydroxymitragynine showed most potent opioid effect on the electrically stimulated contraction (pD2 = 8.38 ± 0.12)	(Horie, Koyama et al., 2005) [121]
	Methanolic and alkaloid extract (100 mg/kg)	Male Swiss mice and Wistar rats	Prolong the latency of nociceptive response in the hot plate test	(Reanmongkol, Keawpradub et al., 2007) [123]
	Mitragynine alkaloid (10 nM–1 μM)	Male Albino guinea pigs	Block the reversible Ca^2+^ channel that activates neurotransmitters	(Matsumoto, Takayama et al., 2005) [122]
	Alkaloid (20 mg/kg), methanolic (200 mg/kg), and aqueous extract (400 mg/kg)	Male Spraque Dawley rats	Both hot plate and tail-flick tests showed prolonged nociceptive responses	(Sabetghadam, Ramanathan et al., 2010) [124]
	Mitragynine alkaloid (100 mg/kg), co-administration of caffeine (25 mg/kg, p.o.) and codeine (3 mg/kg, p.o.)	Male Wistar rats	↑ Latency period in a hot plate test after 30 min	(Botpiboon 2010) [126]
	Mitragynine (2.0 mg/kg) and paynantheine (0.1 mg/kg)	Male wild-type mice (+/+) and μ opioid receptor (MOR) knockout mice (−/−)	Exert analgesic effects predominantly via κ opioid receptors	(Stolt, Schröder et al., 2014) [132]
**Anti** **-depressant**				
**Kratom**	Mitragynine (10 mg/kg and 30 mg/kg)	Male mice from the ICR strain	↓ Corticosterone in forced swim test (FST) and tail suspension test (TST)	(Idayu, Hidayat et al., 2011) [29]
	Aqueous extract (100, 300, and 500 mg/kg)	Male Swiss albino mice	Effects on serotonin or noradrenaline neurotransmissions	(Kumarnsit, Keawpradub et al., 2006) [22]
	Mitragynine or alkaloid extract (20, 40, and 80 mg/kg)	Male Swiss albino mice	↑ Total number of arm entries, rearing frequency and ↓ grooming, and immobility time in the Y-maze test, Alkaloid extract exhibits more potent opioid agonistic effects than mitragynine	(Ammar, Muzaimi et al., 2011) [112]
	Mitragynine (5, 15, 20, and 25 mg/kg)	Male Swiss albino mice	Chronic mitragynine treatment impaired spatial learning and memory	(Ismail, Jayabalan et al., 2017) [113]
	Mitragynine (72.5 mg and 74.9 mg) of Kratom tea or >3 glasses daily	Human	Executive function, memory, and attention were not impaired	(Singh, Narayanan et al., 2019) [36]
**Anti** **-psychotic** **/ Anti** **-dopaminergic**				
**Kratom**	Methanolic extract (75 and 100 mg/kg)	Male Swiss albino mice	Apomorphine-induced cage climbing behavior ↓, ↓ dopamine-induced contractile response	(Vijeepallam, Pandy et al., 2016) [136]

**Table 4:** Interaction of kratom and its active metabolites with the major receptors and modulators.

Kratom and Its Compounds	Receptors/Modulators	Resulting Effects	References
Kratom alkaloid	Inhibition of CYP3A4, CYP2D6, and CYP2C9	Alter drug metabolism	[91]
Mitragynine	Activate GABA_B_ receptor	Anti-depressant activity	[27]
Mitragynine	Antagonize NMDA receptor	Dissociative anesthesia	[110]
Mitragynine	Regulation of the Keap-1/Nrf-2 pathway	Ensure neuroprotection	[115]
Mitragynine	Activation of Nrf2, HO-1, and NQO1	Decrease level of ROS	[115]
Kratom	Binds with nuclear factor kappa B (NF-κB)	Inhibit the release of proinflammatory mediators	[107]
Mitragynine and 7-hydroxymitragynine	Binds with adrenergic-α2 (Aα2R)	Antinociceptive effect	[135]
Mitragynine	Inhibit the enzyme acetylcholinesterase (AChE) receptor	Attenuate Alzheimer’s disease	[148]
Mitragynine	Binds with the HSF-1	Inhibition of the mRNA expression of COX-2	[118]

## Data Availability

All data are enclosed in this review. Additional information may be supplied on request.

## Abbreviations

AChE	Acetylcholinesterase
AD	Alzheimer’s disease
ALT	Alanine aminotransferase
ASE	Accelerated solvent extraction
AST	Aspartate aminotransferase
BBB	Blood–brain barrier
CAT	Catalase
COX-2	Cyclooxygenase-2
CUPRAC	Cupric ion reducing antioxidant capacity
CYPs	Cytochromes P50
ED_50_	Median effective dose
ESI	Electrospray ionization
FRAP	Ferric reducing ability of plasma
fEPSP	Field excitatory postsynaptic potentials
HSP	Heat shock proteins
HSF	Heat shock Factors
HT2A	Hydroxy-Tryptamine receptor
IC_50_	Half maximal inhibitory concentration
I. P	Intraperitoneal
Keap1	Kelch-like ECH-Associating protein 1
Kg	Kilogram
LD_50_	Median lethal dose
LPS	Lipopolysaccharides
LTP	Long-term potentiation
MAO	Monoamine oxidase
Mg	Milligram
µg	Microgram
MIC	Minimum inhibitory concentration
Nrf2	Nuclear factor erythroid 2-related factor 2
Nm	Nanometer
PD	Parkinson’s disease
P. O	Per Oral
QTOF-MS	Quadrupole time-of-flight mass spectrometry
SOD	Superoxide dismutase
TPC	Total phenolic content
TFC	Total flavonoid content
UDP	Uridine diphosphate
UGT	UDP-glucuronosyl transferase
UHPLC	Ultra high-performance liquid chromatography
*w*/*w*	Weight for weight

## References

[1] Butler M.S. (2004). The role of natural product chemistry in drug discovery. J. Nat. Prod..

[2] Newman D.J., Cragg G.M. (2012). Natural products as sources of new drugs over the 30 years from 1981 to 2010. J. Nat. Prod..

[3] Koehn F.E., Carter G.T. (2005). The evolving role of natural products in drug discovery. Nat. Rev. Drug Discov..

[4] Patwardhan B., Mashelkar R.A. (2009). Traditional medicine-inspired approaches to drug discovery: Can Ayurveda show the way forward?. Drug Discov. Today.

[5] Arndt T., Claussen U., Güssregen B., Schröfel S., Stürzer B., Werle A., Wolf G. (2011). Kratom alkaloids and O-desmethyltramadol in urine of a “Krypton” herbal mixture consumer. Forensic Sci. Int..

[6] White C.M. (2018). Pharmacologic and clinical assessment of kratom. Bull. Am. Soc. Hosp. Pharm..

[7] Gong F., Gu H.P., Xu Q.T., Kang W.Y. (2012). Genus *Mitragyna*: Ethnomedicinal uses and pharmacological studies. Phytopharmacology.

[8] Rech M.A., Donahey E., Dziedzic J.M.C., Oh L., Greenhalgh E. (2015). New drugs of abuse. Pharmacotherapy. J. Hum. Pharmacol. Drug Ther..

[9] Vicknasingam B., Narayanan S., Beng G.T., Mansor S.M. (2010). The informal use of ketum (*Mitragyna speciosa*) for opioid withdrawal in the northern states of peninsular Malaysia and implications for drug substitution therapy. Int. J. Drug Policy.

[10] Hassan Z., Muzaimi M., Navaratnam V., Yusoff N.H., Suhaimi F.W., Vadivelu R., Vicknasingam B.K., Amato D., von Hörsten S., Ismail N.I. (2013). From Kratom to mitragynine and its derivatives: Physiological and behavioural effects related to use, abuse, and addiction. Neurosci. Biobehav. Rev..

[11] Eisenman S.W. (2014). The botany of *Mitragyna speciosa* (Korth.) Havil. and related species. Kratom and Other Mitragynines: The Chemistry and Pharmacology of Opioids from a Non-Opium Source.

[12] Warner M.L., Kaufman N.C., Grundmann O. (2016). The pharmacology and toxicology of kratom: From traditional herb to drug of abuse. Int. J. Leg. Med..

[13] Adkins J.E., Boyer E.W., McCurdy C.R. (2011). *Mitragyna speciosa*, a psychoactive tree from Southeast Asia with opioid activity. Curr. Top. Med. Chem..

[14] Jaipaew J., Padungchareon T., Sukrong S. (2018). PCR-reverse dot blot of the nucleotide signature sequences of matK for the identification of *Mitragyna speciosa*, a narcotic species. Plant Gene.

[15] Tungphatthong C., Urumarudappa S.K.J., Awachai S., Sooksawate T., Sukrong S. (2021). Differentiation of *Mitragyna speciosa*, a narcotic plant, from allied Mitragyna species using DNA barcoding-high-resolution melting (Bar-HRM) analysis. Sci. Rep..

[16] Parthasarathy S., Bin Azizi J., Ramanathan S., Ismail S., Sasidharan S., Said M.I.M., Mansor S.M. (2009). Evaluation of antioxidant and antibacterial activities of aqueous, methanolic and alkaloid extracts from *Mitragyna speciosa* (Rubiaceae family) leaves. Molecules.

[17] Meireles V., Rosado T., Barroso M., Soares S., Gonçalves J., Luís A., Caramelo D., Simão A.Y., Fernández N., Duarte A.P. (2019). *Mitragyna speciosa*: Clinical, toxicological aspects and analysis in biological and non-biological samples. Medicines.

[18] Yuniarti R., Nadia S., Alamanda A., Zubir M., Syahputra R.A., Nizam M. (2020). Characterization, phytochemical screenings and antioxidant activity test of kratom leaf ethanol extract (*Mitragyna speciosa* Korth) using DPPH method. J. Phys. Conf. Ser. IOP Publ..

[19] Srichana K., Janchawee B., Prutipanlai S., Raungrut P., Keawpradub N. (2015). Effects of mitragynine and a crude alkaloid extract derived from *Mitragyna speciosa* Korth. on permethrin elimination in rats. Pharmaceutics.

[20] Goh Y.S., Karunakaran T., Murugaiyah V., Santhanam R., Abu Bakar M.H., Ramanathan S. (2021). Accelerated solvent extractions (ASE) of *Mitragyna speciosa* Korth. (Kratom) leaves: Evaluation of its cytotoxicity and antinociceptive activity. Molecules.

[21] Mossadeq W.S., Sulaiman M., Mohamad T.T., Chiong H., Zakaria Z., Jabit M., Baharuldin M., Israf D. (2009). Anti-inflammatory and antinociceptive effects of *Mitragyna speciosa* Korth methanolic extract. Med. Princ. Pract..

[22] Kumarnsit E., Keawpradub N., Nuankaew W. (2006). Acute and long-term effects of alkaloid extract of *Mitragyna speciosa* on food and water intake and body weight in rats. Fitoterapia.

[23] Watanabe K., Yano S., Horie S., Yamamoto L.T. (1997). Inhibitory effect of mitragynine, an alkaloid with analgesic effect from Thai medicinal plant *Mitragyna speciosa*, on electrically stimulated contraction of isolated guinea-pig ileum through the opioid receptor. Life Sci..

[24] Pathak L., Agrawal Y., Dhir A. (2013). Natural polyphenols in the management of major depression. Expert Opin. Investig. Drugs.

[25] Carpenter J.M., Criddle C.A., Craig H.K., Ali Z., Zhang Z., Khan I.A., Sufka K.J. (2016). Comparative effects of *Mitragyna speciosa* extract, mitragynine, and opioid agonists on thermal nociception in rats. Fitoterapia.

[26] Kruegel A.C., Uprety R., Grinnell S.G., Langreck C., Pekarskaya E.A., Le Rouzic V., Ansonoff M., Gassaway M.M., Pintar J.E., Pasternak G.W. (2019). 7-Hydroxymitragynine is an active metabolite of mitragynine and a key mediator of its analgesic effects. ACS Cent. Sci..

[27] Kumarnsit E., Keawpradub N., Nuankaew W. (2007). Effect of *Mitragyna speciosa* aqueous extract on ethanol withdrawal symptoms in mice. Fitoterapia.

[28] Apryani E., Hidayat M.T., Moklas M., Fakurazi S., Idayu N.F. (2010). Effects of mitragynine from *Mitragyna speciosa* Korth leaves on working memory. J. Ethnopharmacol..

[29] Idayu N.F., Hidayat M.T., Moklas M., Sharida F., Raudzah A.N., Shamima A., Apryani E. (2011). Antidepressant-like effect of mitragynine isolated from *Mitragyna speciosa* Korth in mice model of depression. Phytomedicine.

[30] Obeng S., Kamble S.H., Reeves M.E., Restrepo L.F., Patel A., Behnke M., Chear N.J.Y., Ramanathan S., Sharma A., León F. (2019). Investigation of the adrenergic and opioid binding affinities, metabolic stability, plasma protein binding properties, and functional effects of selected indole-based kratom alkaloids. J. Med. Chem..

[31] Johnson L.E., Balyan L., Magdalany A., Saeed F., Salinas R., Wallace S., Veltri C.A., Swogger M.T., Walsh Z., Grundmann O. (2020). Focus: Plant-based Medicine and Pharmacology: The Potential for Kratom as an Antidepressant and Antipsychotic. Yale J. Biol. Med..

[32] Ahmad I., Prabowo W.C., Arifuddin M., Fadraersada J., Indriyanti N., Herman H., Purwoko R.Y., Nainu F., Rahmadi A., Paramita S. (2022). *Mitragyna* species as pharmacological agents: From abuse to promising pharmaceutical products. Life.

[33] Vijeepallam K., Pandy V., Murugan D.D., Naidu M. (2019). Methanolic extract of *Mitragyna speciosa* Korth leaf inhibits ethanol seeking behaviour in mice: Involvement of antidopaminergic mechanism. Metab. Brain Dis..

[34] Salleh N.A.S.M., Halim S., Ridzuan P.M., Uzid M.M., Ramli M.D. (2021). The Potential Role of Neuroprotective Effects of Kratom (*Mitragyna speciosa*) On Brain Aging. J. Cell. Mol. Anesth..

[35] Aznal A.N.Z., Hazalin N.A.M.N., Hassan Z., Mat N.H., Chear N.J.-Y., Teh L.K., Salleh M.Z., Suhaimi F.W. (2022). Adolescent kratom exposure affects cognitive behaviours and brain metabolite profiles in Sprague-Dawley rats. Front. Pharmacol..

[36] Singh D., Narayanan S., Müller C.P., Vicknasingam B., Yücel M., Ho E.T.W., Hassan Z., Mansor S.M. (2019). Long-Term Cognitive Effects of Kratom (*Mitragyna speciosa* Korth.) Use. J. Psychoact. Drugs.

[37] Flores-Bocanegra L., Raja H.A., Graf T.N., Augustinović M., Wallace E.D., Hematian S., Kellogg J.J., Todd D.A., Cech N.B., Oberlies N.H. (2020). The Chemistry of Kratom [*Mitragyna speciosa*]: Updated Characterization Data and Methods to Elucidate Indole and Oxindole Alkaloids. J. Nat. Prod..

[38] Suwanlert S. (1975). A study of kratom eaters in Thailand. Bull. Narc..

[39] Ahmad K., Aziz Z. (2012). *Mitragyna speciosa* use in the northern states of Malaysia: A cross-sectional study. J. Ethnopharmacol..

[40] Cinosi E., Martinotti G., Simonato P., Singh D., Demetrovics Z., Roman-Urrestarazu A., Bersani F.S., Vicknasingam B., Piazzon G., Li J.-H. (2015). Following “the roots” of Kratom (*Mitragyna speciosa*): The evolution of an enhancer from a traditional use to increase work and productivity in Southeast Asia to a recreational psychoactive drug in western countries. BioMed Res. Int..

[41] Ruck C. (2021). Mushrooms, Myth and Mithras: The Drug Cult that Civilized Europe.

[42] Tanguay P. (2011). Kratom in Thailand: Decriminalisation and Community Control?.

[43] Saingam D., Assanangkornchai S., Geater A.F., Balthip Q. (2013). Pattern and consequences of krathom (*Mitragyna speciosa* Korth.) use among male villagers in southern Thailand: A qualitative study. Int. J. Drug Policy.

[44] Singh D., Narayanan S., Vicknasingam B. (2016). Traditional and non-traditional uses of Mitragynine (Kratom): A survey of the literature. Brain Res. Bull..

[45] Khalil S., Abdullah S.A.J., Ahmad R. (2020). Enforcement status of the poison act 1952 against offences related to kratom (*Mitragyna speciosa* korth) misuse in Malaysia. UUM J. Leg. Stud..

[46] Swogger M.T., Hart E., Erowid F., Erowid E., Trabold N., Yee K., Parkhurst K.A., Priddy B.M., Walsh Z. (2015). Experiences of kratom users: A qualitative analysis. J. Psychoact. Drugs.

[47] Assanangkornchai S., Muekthong A., Sam-Angsri N., Pattanasattayawong U. (2007). The use of *Mitragynine speciosa* (“Krathom”), an addictive plant, in Thailand. Subst. Use Misuse.

[48] Grundmann O. (2017). Patterns of Kratom use and health impact in the US-Results from an online survey. Drug Alcohol Depend..

[49] Hillebrand J., Olszewski D., Sedefov R. (2010). Legal highs on the Internet. Subst. Use Misuse.

[50] Schmidt M.M., Sharma A., Schifano F., Feinmann C. (2011). “Legal highs” on the net-Evaluation of UK-based Websites, products and product information. Forensic Sci. Int..

[51] Feng L.-Y., Battulga A., Han E., Chung H., Li J.-H. (2017). New psychoactive substances of natural origin: A brief review. J. Food Drug Anal..

[52] Mustafa A., Trevino L.M., Turner C. (2012). Pressurized hot ethanol extraction of carotenoids from carrot by-products. Molecules.

[53] Mohamed H.M. (2015). Green, environment-friendly, analytical tools give insights in pharmaceuticals and cosmetics analysis. TrAC Trends Anal. Chem..

[54] Techasakul S., Chimnoi N., Khunnawutm N., Feungfuloy P., Chatrewong K. (2013). Facile isolation and purification of thailandine, a biologically active oxoaporphine alkaloid, from *Stephania venosa* leaves using ion-pair liquid-liquid extraction. Res. J. Med. Plant.

[55] Fatima N., Tapondjou L.A., Lontsi D., Sondengam B., Rahman A.U., Choudhary M.I. (2002). Quinovic acid glycosides from Mitragyna stipulosa-first examples of natural inhibitors of snake venom phosphodiesterase I. Nat. Prod. Lett..

[56] Asase A., Kokubun T., Grayer R.J., Kite G., Simmonds M.S.J., Oteng-Yeboah A.A., Odamtten G.T. (2008). Chemical constituents and antimicrobial activity of medicinal plants from Ghana: Cassia sieberiana, Haematostaphis barteri, Mitragyna inermis and Pseudocedrela kotschyi. Phytother. Res..

[57] Phongprueksapattana S., Putalun W., Keawpradub N., Wungsintaweekul J. (2008). *Mitragyna speciosa*: Hairy root culture for triterpenoid production and high yield of mitragynine by regenerated plants. Z. Naturforsch. C J. Biosci..

[58] Ponglux D., Wongseripipatana S., Takayama H., Kikuchi M., Kurihara M., Kitajima M., Aimi N., Sakai S.-I. (1994). A New Indole Alkaloid, 7 alpha-Hydroxy-7H-mitragynine, from *Mitragyna speciosa* in Thailand. Planta Med..

[59] León F., Habib E., Adkins J.E., Furr E.B., McCurdy C.R., Cutler S.J. (2009). Phytochemical characterization of the leaves of *Mitragyna speciosa* grown in U.S.A. Nat. Prod. Commun..

[60] Jaleel C.A., Gopi R., Kishorekumar A., Manivannan P., Sankar B., Panneerselvam R. (2008). Interactive effects of triadimefon and salt stress on antioxidative status and ajmalicine accumulation in *Catharanthus roseus*. Acta Physiol. Plant..

[61] Gajalakshmi S., Vijayalakshmi S., Devi R.V. (2013). Pharmacological activities of Catharanthus roseus: A perspective review. Int. J. Pharma Bio Sci..

[62] Duwiejua M., Woode E., Obiri D. (2002). Pseudo-akuammigine, an alkaloid from *Picralima nitida* seeds, has anti-inflammatory and analgesic actions in rats. J. Ethnopharmacol..

[63] Trager W., Lee C.M., Phillipson J., Haddock R., Dwuma-Badu D., Beckett A. (1968). Configurational analysis of rhynchophylline-type oxindole alkaloids. The absolute configuration of ciliaphylline, rhynchociline, specionoxeine, isospecionoxeine, rotundifoline and isorotundifoline. Tetrahedron.

[64] Roquebert J., Demichel P. (1984). Inhibition of the alpha 1 and alpha 2-adrenoceptor-mediated pressor response in pithed rats by raubasine, tetrahydroalstonine and akuammigine. Eur. J. Pharmacol..

[65] Chen L.-L., Song J.-X., Lu J.-H., Yuan Z.-W., Liu L.-F., Durairajan S.S.K., Li M. (2014). Corynoxine, a Natural Autophagy Enhancer, Promotes the Clearance of Alpha-Synuclein via Akt/mTOR Pathway. J. Neuroimmune Pharmacol..

[66] Gutierrez-Salmean G., Ciaraldi T.P., Nogueira L., Barboza J., Taub P.R., Hogan M.C., Henry R.R., Meaney E., Villarreal F., Ceballos G. (2014). Effects of (−)-epicatechin on molecular modulators of skeletal muscle growth and differentiation. J. Nutr. Biochem..

[67] Escandón R.A., del Campo M., López-Solis R., Obreque-Slier E., Toledo H. (2016). Antibacterial effect of kaempferol and (−)-epicatechin on *Helicobacter pylori*. Eur. Food Res. Technol..

[68] Matsumoto K., Hatori Y., Murayama T., Tashima K., Wongseripipatana S., Misawa K., Kitajima M., Takayama H., Horie S. (2006). Involvement of μ-opioid receptors in antinociception and inhibition of gastrointestinal transit induced by 7-hydroxymitragynine, isolated from Thai herbal medicine *Mitragyna speciosa*. Eur. J. Pharmacol..

[69] Shukla A., Srinivasan B.P. (2012). 16,17-Dihydro-17b-hydroxy isomitraphylline alkaloid as an inhibitor of DPP-IV, and its effect on incretin hormone and β-cell proliferation in diabetic rat. Eur. J. Pharm Sci..

[70] García R., Cayunao C., Bocic R., Backhouse N., Delporte C., Zaldivar M., Erazo S. (2005). Antimicrobial activity of isopteropodine. Z. Naturforsch. C J. Biosci..

[71] Wang X.L., Zhang L.M., Hua Z. (1994). Blocking effect of rhynchophylline on calcium channels in isolated rat ventricular myocytes. Zhongguo Yao Li Xue Bao.

[72] Kocialski A.B., Marozzi F.J., Malone M.H. (1972). Effects of certain nonsteroid anti-inflammatory drugs, tolbutamide, and tetrahydroalstonine on blood glucose and carrageen in-induced pedal edema in rats. J. Pharm. Sci..

[73] Kang T.-H., Murakami Y., Matsumoto K., Takayama H., Kitajima M., Aimi N., Watanabe H. (2002). Rhynchophylline and isorhynchophylline inhibit NMDA receptors expressed in Xenopus oocytes. Eur. J. Pharmacol..

[74] Philipp A.A., Wissenbach D.K., Weber A.A., Zapp J., Maurer H.H. (2011). Metabolism studies of the Kratom alkaloids mitraciliatine and isopaynantheine, diastereomers of the main alkaloids mitragynine and paynantheine, in rat and human urine using liquid chromatography-linear ion trap-mass spectrometry. J Chromatogr. B Analyt. Technol. Biomed Life Sci..

[75] Kruegel A.C., Gassaway M.M., Kapoor A., Váradi A., Majumdar S., Filizola M., Javitch J.A., Sames D. (2016). Synthetic and Receptor Signaling Explorations of the *Mitragyna* Alkaloids: Mitragynine as an Atypical Molecular Framework for Opioid Receptor Modulators. J. Am. Chem. Soc..

[76] Joshi B., Taylor W. (1963). Structure of mitragynine (9-methoxycorynantheidine). Chem. Ind..

[77] Zacharias D.E., Rosenstein R.D., Jeffrey G.A. (1965). The structure of mitragynine hydroiodide. Acta Crystallogr..

[78] Takayama H., Ishikawa H., Kurihara M., Kitajima M., Aimi N., Ponglux D., Koyama F., Matsumoto K., Moriyama T., Yamamoto L.T. (2002). Studies on the synthesis and opioid agonistic activities of mitragynine-related indole alkaloids: Discovery of opioid agonists structurally different from other opioid ligands. J. Med. Chem..

[79] Sharma A., Kamble S.H., Leon F., Chear N.J.Y., King T.I., Berthold E.C., Ramanathan S., McCurdy C.R., Avery B.A. (2019). Simultaneous quantification of ten key Kratom alkaloids in *Mitragyna speciosa* leaf extracts and commercial products by ultra-performance liquid chromatography-tandem mass spectrometry. Drug Test. Anal..

[80] Ellis C.R., Racz R., Kruhlak N.L., Kim M.T., Zakharov A.V., Southall N., Hawkins E.G., Burkhart K., Strauss D.G., Stavitskaya L. (2020). Evaluating kratom alkaloids using PHASE. PLoS ONE.

[81] Gutridge A.M., Robins M.T., Cassell R.J., Uprety R., Mores K.L., Ko M.J., Pasternak G.W., Majumdar S., van Rijn R.M. (2020). G protein-biased kratom-alkaloids and synthetic carfentanil-amide opioids as potential treatments for alcohol use disorder. Br. J. Pharmacol..

[82] Chear N.J.-Y., León F., Sharma A., Kanumuri S.R.R., Zwolinski G., Abboud K.A., Singh D., Restrepo L.F., Patel A., Hiranita T. (2021). Exploring the Chemistry of Alkaloids from Malaysian *Mitragyna speciosa* (Kratom) and the Role of Oxindoles on Human Opioid Receptors. J. Nat. Prod..

[83] Houghton P.J., Said I.M. (1986). 3-dehydromitragynine: An alkaloid from *Mitragyna speciosa*. Phytochemistry.

[84] Houghton P.J., Latiff A., Said I.M. (1991). Alkaloids from *Mitragyna speciosa*. Phytochemistry.

[85] Limsuwanchote S., Wungsintaweekul J., Keawpradub N., Putalun W., Morimoto S., Tanaka H. (2014). Development of indirect competitive ELISA for quantification of mitragynine in Kratom (*Mitragyna speciosa* (Roxb.) Korth.). Forensic Sci. Int..

[86] Ramanathan S., León F., Chear N.J., Yusof S.R., Murugaiyah V., McMahon L.R., McCurdy C.R. (2021). Kratom (*Mitragyna speciosa* Korth.): A description on the ethnobotany, alkaloid chemistry, and neuropharmacology. Stud. Nat. Prod. Chem..

[87] Manwill P.K., Flores-Bocanegra L., Khin M., Raja H.A., Cech N.B., Oberlies N.H., Todd D.A. (2022). Kratom (*Mitragyna speciosa*) Validation: Quantitative Analysis of Indole and Oxindole Alkaloids Reveals Chemotypes of Plants and Products. Planta Medica.

[88] Wang M., Carrell E.J., Ali Z., Avula B., Avonto C., Parcher J.F., Khan I.A. (2014). Comparison of three chromatographic techniques for the detection of mitragynine and other indole and oxindole alkaloids in *Mitragyna speciosa* (kratom) plants. J. Sep. Sci..

[89] Azizi J., Ismail S., Mordi M.N., Ramanathan S., Said M.I.M., Mansor S.M. (2010). In vitro and in vivo effects of three different *Mitragyna speciosa* Korth leaf extracts on phase II drug metabolizing enzymes—Glutathione transferases (GSTs). Molecules.

[90] Raffa R.B. (2014). Kratom and Other Mitragynines: The Chemistry and Pharmacology of Opioids from a Non-Opium Source.

[91] Veeramohan R., Azizan K.A., Aizat W.M., Goh H.-H., Mansor S.M., Yusof N.S.M., Baharum S.N., Ng C.L. (2018). Metabolomics data of *Mitragyna speciosa* leaf using LC-ESI-TOF-MS. Data Brief.

[92] Trakulsrichai S., Sathirakul K., Auparakkitanon S., Krongvorakul J., Sueajai J., Noumjad N., Sukasem C., Wananukul W. (2015). Pharmacokinetics of mitragynine in man. Drug Des. Dev. Ther..

[93] Smith K.E., Rogers J.M., Dunn K.E., Grundmann O., McCurdy C.R., Schriefer D., Epstein D.H. (2022). Searching for a Signal: Self-Reported Kratom Dose-Effect Relationships Among a sample of US adults with regular Kratom use histories. Front. Pharmacol..

[94] Manda V.K., Avula B., Ali Z., Khan I.A., Walker L.A., Khan S.I. (2014). Evaluation of in vitro absorption, distribution, metabolism, and excretion (ADME) properties of mitragynine, 7-hydroxymitragynine, and mitraphylline. Planta Med..

[95] Harizal S., Mansor S., Hasnan J., Tharakan J., Abdullah J. (2010). Acute toxicity study of the standardized methanolic extract of *Mitragyna speciosa* Korth in rodents. J. Ethnopharmacol..

[96] Ilmie M.U., Jaafar H., Mansor S.M., Abdullah J.M. (2015). Subchronic toxicity study of standardized methanolic extract of *Mitragyna speciosa* Korth in Sprague-Dawley Rats. Front. Neurosci..

[97] Saidin N.A., Randall T., Takayama H., Holmes E., Gooderham N.J. (2008). Malaysian Kratom, a phyto-pharmaceutical of abuse: Studies on the mechanism of its cytotoxicity. Toxicology.

[98] Rusli N., Amanah A., Kaur G., Adenan M.I., Sulaiman S.F., Wahab H.A., Tan M.L. (2019). The inhibitory effects of mitragynine on P-glycoprotein in vitro. Naunyn-Schmiedeberg’s Arch. Pharmacol..

[99] Azizi J., Ismail S., Mansor S.M. (2013). *Mitragyna speciosa* Korth leaves extracts induced the CYP450 catalyzed aminopyrine-N-demethylase (APND) and UDP-glucuronosyl transferase (UGT) activities in male Sprague-Dawley rat livers. Drug Metab. Drug Interact..

[100] Kong W.M., Chik Z., Ramachandra M., Subramaniam U., Aziddin R.E.R., Mohamed Z. (2011). Evaluation of the effects of *Mitragyna speciosa* alkaloid extract on cytochrome P450 enzymes using a high throughput assay. Molecules.

[101] Kamble S.H., Sharma A., King T.I., León F., McCurdy C.R., Avery B.A. (2019). Metabolite profiling and identification of enzymes responsible for the metabolism of mitragynine, the major alkaloid of *Mitragyna speciosa* (kratom). Xenobiotica.

[102] Uno Y., Uehara S., Murayama N., Yamazaki H. (2018). Cytochrome P450 1A1, 2C9, 2C19, and 3A4 polymorphisms account for interindividual variability of toxicological drug metabolism in cynomolgus macaques. Chem. Res. Toxicol..

[103] Ismail S., Mansor S., Hanapi N. (2013). Inhibitory effect of mitragynine on human cytochrome P450 enzyme activities. Pharmacogn. Res..

[104] Showande S.J., Fakeye T.O., Kajula M., Hokkanen J., Tolonen A. (2019). Potential inhibition of major human cytochrome P450 isoenzymes by selected tropical medicinal herbs—Implication for herb–drug interactions. Food Sci. Nutr..

[105] Ulbricht C., Costa D., Dao J., Isaac R., LeBlanc Y.C., Rhoades J., Windsor R.C. (2013). An evidence-based systematic review of kratom (*Mitragyna speciosa*) by the Natural Standard Research Collaboration. J. Diet. Suppl..

[106] Hughes R.L. (2019). Fatal combination of mitragynine and quetiapine–a case report with discussion of a potential herb-drug interaction. Forensic Sci. Med. Pathol..

[107] Fluyau D., Revadigar N. (2017). Biochemical benefits, diagnosis, and clinical risks evaluation of kratom. Front. Psychiatry.

[108] Grewal K.S. (1932). Observations OX the Pharmacology of Mitragynine. J. Pharmacol..

[109] Mohammad Yusoff N.H., Mansor S.M., Visweswaran N., Muller C.P., Hassan Z. GABA_B_ receptor system modulates mitragynine-induced conditioned place preference in rats. Proceedings of the 14th Meeting of the Asian-Pacific Society for Neurochemistry.

[110] Effendy M.A., Yunusa S., Mat N.H., Has A.T.C., Müller C.P., Hassan Z. (2023). The role of AMPA and NMDA receptors in mitragynine effects on hippocampal synaptic plasticity. Behav. Brain Res..

[111] Kruegel A.C., Grundmann O. (2018). The medicinal chemistry and neuropharmacology of kratom: A preliminary discussion of a promising medicinal plant and analysis of its potential for abuse. Neuropharmacology.

[112] Ammar I.H., Muzaimi M., Sharif M.M. (2011). The effects on motor behaviour and short-term memory tasks in mice following an acute administration of *Mitragyna speciosa* alkaloid extract and mitragynine. J. Med. Plants Res..

[113] Ismail N.I.W., Jayabalan N., Mansor S.M., Müller C.P., Muzaimi M. (2017). Chronic mitragynine (kratom) enhances punishment resistance in natural reward seeking and impairs place learning in mice. Addict. Biol..

[114] Senik M., Mansor S., Rammes G., Tharakan J., Abdullah J. (2012). *Mitragyna speciosa* Korth standardized methanol extract induced short-term potentiation of CA1 subfield in rat hippocampal slices. J. Med. Plants Res..

[115] Raymond-Hamet A. (1950). Les alcaloïdes du *Mitragyna speciosa* Korthals [The alkaloids of *Mitragyna speciosa* Korthals–French]. Ann. Pharm. Françaises.

[116] Federico A., Morgillo F., Tuccillo C., Ciardiello F., Loguercio C. (2007). Chronic inflammation and oxidative stress in human carcinogenesis. Int. J. Cancer.

[117] Hussain S.P., Harris C.C. (2007). Inflammation and cancer: An ancient link with novel potentials. Int. J. Cancer.

[118] Utar Z., Majid M.I.A., Adenan M.I., Jamil M.F.A., Lan T.M. (2011). Mitragynine inhibits the COX-2 mRNA expression and prostaglandin E2 production induced by lipopolysaccharide in RAW264.7 macrophage cells. J. Ethnopharmacol..

[119] Otero-Losada M., Capani F., Perez Lloret S. (2020). Neuroprotection—New Approaches and Prospects.

[120] Vermaire D.J., Skaer D., Tippets W. (2019). Kratom and general anesthesia: A case report and review of the literature. AA Pract..

[121] Horie S., Koyama F., Takayama H., Ishikawa H., Aimi N., Ponglux D., Matsumoto K., Murayama T. (2005). Indole alkaloids of a Thai medicinal herb, *Mitragyna speciosa*, that has opioid agonistic effect in guinea-pig ileum. Planta Medica.

[122] Matsumoto K., Takayama H., Ishikawa H., Aimi N., Ponglux D., Watanabe K., Horie S. (2005). Partial agonistic effect of 9-hydroxycorynantheidine on mu-opioid receptor in the guinea-pig ileum. Life Sci..

[123] Reanmongkol W., Keawpradub N., Sawangjaroen K. (2007). Effects of the extracts from *Mitragyna speciosa* Korth. leaves on analgesic and behavioral activities in experimental animals. Songklanakarin J. Sci. Technol..

[124] Sabetghadam A., Ramanathan S., Mansor S.M. (2010). The evaluation of antinociceptive activity of alkaloid, methanolic, and aqueous extracts of Malaysian *Mitragyna speciosa* Korth leaves in rats. Pharmacogn. Res..

[125] Idid S., Saad L., Yaacob H., Shahimi M. (1998). Evaluation of analgesia induced by mitragynine, morphine and paracetamol on mice. ASEAN Rev. Biodivers. Environ. Conserv..

[126] Botpiboon O. (2010). Effects of Caffeine and Codeine on Pharmacokinetics and Antinociceptive Activity of Alkaloid Extract from Leaves of Kratom (*Mitragyna speciosa* Korth.). Ph.D. Thesis.

[127] Matsumoto K., Mizowaki M., Suchitra T., Takayama H., Sakai S.-I., Aimi N., Watanabe H. (1996). Antinociceptive action of mitragynine in mice: Evidence for the involvement of supraspinal opioid receptors. Life Sci..

[128] Boyer E.W., Babu K.M., Adkins J.E., McCurdy C.R., Halpern J.H. (2008). Self-treatment of opioid withdrawal using kratom (*Mitragynia speciosa* korth). Addiction.

[129] Takayama H. (2004). Chemistry and pharmacology of analgesic indole alkaloids from the rubiaceous plant, *Mitragyna speciosa*. Chem. Pharm. Bull..

[130] Thongpradichote S., Matsumoto K., Tohda M., Takayama H., Aimi N., Sakai S.-I., Watanabe H. (1998). Identification of opioid receptor subtypes in antinociceptive actions of supraspinally-admintstered mitragynine in mice. Life Sci..

[131] Yamamoto L.T., Horie S., Takayama H., Aimi N., Sakai S.-I., Yano S., Shan J., Pang P.K., Ponglux D., Watanabe K. (1999). Opioid receptor agonistic characteristics of mitragynine pseudoindoxyl in comparison with mitragynine derived from Thai medicinal plant *Mitragyna speciosa*. Gen. Pharmacol. Vasc. Syst..

[132] Stolt A.-C., Schröder H., Neurath H., Grecksch G., Höllt V., Meyer M.R., Maurer H.H., Ziebolz N., Havemann-Reinecke U., Becker A. (2014). Behavioral and neurochemical characterization of kratom (*Mitragyna speciosa*) extract. Psychopharmacology.

[133] Hemby S.E., McIntosh S., Leon F., Cutler S.J., McCurdy C.R. (2019). Abuse liability and therapeutic potential of the *Mitragyna speciosa* (kratom) alkaloids mitragynine and 7-hydroxymitragynine. Addict. Biol..

[134] Havemann-Reinecke U. (2011). P01-50-Kratom and alcohol dependence: Clinical symptoms, withdrawal treatment and pharmacological mechanisms-A case report. Eur. Psychiatry.

[135] Obeng S., Leon F., Patel A., Gonzalez J.D.Z., Da Silva L.C., Restrepo L.F., Gamez-Jimenez L.R., Ho N.P., Calvache M.P.G., Pallares V.L. (2022). Interactive Effects of µ-Opioid and Adrenergic-α (2) Receptor Agonists in Rats: Pharmacological Investigation of the Primary Kratom Alkaloid Mitragynine and Its Metabolite 7-Hydroxymitragynine. J. Pharmacol. Exp. Ther..

[136] Vijeepallam K., Pandy V., Kunasegaran T., Murugan D.D., Naidu M. (2016). *Mitragyna speciosa* leaf extract exhibits antipsychotic-like effect with the potential to alleviate positive and negative symptoms of psychosis in mice. Front. Pharmacol..

[137] Lu J., Wei H., Wu J., Jamil M.F.A., Tan M.L., Adenan M.I., Wong P., Shim W. (2014). Evaluation of the cardiotoxicity of mitragynine and its analogues using human induced pluripotent stem cell-derived cardiomyocytes. PLoS ONE.

[138] Sandager M., Nielsen N.D., Stafford G.I., van Staden J., Jäger A.K. (2005). Alkaloids from *Boophane disticha* with affinity to the serotonin transporter in rat brain. J. Ethnopharmacol..

[139] Elgorashi E.E., Stafford G.I., Jäger A.K., Van Staden J. (2006). Inhibition of [3H] citalopram binding to the rat brain serotonin transporter by Amaryllidaceae alkaloids. Planta Medica.

[140] Neergaard J.S., Andersen J., Pedersen M.E., Stafford G.I., Van Staden J., Jäger A.K. (2009). Alkaloids from *Boophone disticha* with affinity to the serotonin transporter. S. Afr. J. Bot..

[141] Kong L., Cheng C.H., Tan R. (2004). Inhibition of MAO A and B by some plant-derived alkaloids, phenols and anthraquinones. J. Ethnopharmacol..

[142] Oboh G., Oyeleye S., Ademiluyi A. (2017). The food and medicinal values of indigenous leafy vegetables. Afr. Veg. Forum.

[143] Nwanna E., Oyeleye S., Ogunsuyi O., Oboh G., Boligon A., Athayde M. (2016). In vitro neuroprotective properties of some commonly consumed green leafy vegetables in Southern Nigeria. NFS J..

[144] Oboh G., Akinyemi A.J., Adeleye B., Oyeleye S.I., Ogunsuyi O.B., Ademosun A.O., Ademiluyi A.O., Boligon A.A. (2016). Polyphenolic compositions and in vitro angiotensin-I-converting enzyme inhibitory properties of common green leafy vegetables: A comparative study. Food Sci. Biotechnol..

[145] Oboh G., Ogunruku O.O., Oyeleye S.I., Olasehinde T.A., Ademosun A.O., Boligon A.A. (2017). Phenolic extracts from Clerodendrum volubile leaves inhibit cholinergic and monoaminergic enzymes relevant to the management of some neurodegenerative diseases. J. Diet. Suppl..

[146] Lühr S., Vilches-Herrera M., Fierro A., Ramsay R.R., Edmondson D.E., Reyes-Parada M., Cassels B.K., Iturriaga-Vásquez P. (2010). 2-Arylthiomorpholine derivatives as potent and selective monoamine oxidase B inhibitors. Bioorganic Med. Chem..

[147] Chen L., Fei S., Olatunji O.J. (2022). LC/ESI/TOF-MS Characterization, Anxiolytic and Antidepressant-like Effects of *Mitragyna speciosa* Korth Extract in Diabetic Rats. Molecules.

[148] Innok W., Hiranrat A., Chana N., Rungrotmongkol T., Kongsune P. (2021). In silico and in vitro anti-AChE activity investigations of constituents from *Mytragyna speciosa* for Alzheimer’s disease treatment. J. Comput.-Aided Mol. Des..

[149] Tsuji M., Takeuchi T., Miyagawa K., Ishii D., Imai T., Takeda K., Kitajima M., Takeda H. (2014). Yokukansan, a traditional Japanese herbal medicine, alleviates the emotional abnormality induced by maladaptation to stress in mice. Phytomedicine.

[150] Neef D.W., Jaeger A.M., Thiele D.J. (2011). Heat shock transcription factor 1 as a therapeutic target in neurodegenerative diseases. Nat. Rev. Drug Discov..

[151] Gomez-Pastor R., Burchfiel E.T., Thiele D.J. (2018). Regulation of heat shock transcription factors and their roles in physiology and disease. Nat. Rev. Mol. Cell Biol..

[152] Steinkraus K.A., Smith E.D., Davis C., Carr D., Pendergrass W.R., Sutphin G.L., Kennedy B.K., Kaeberlein M. (2008). Dietary restriction suppresses proteotoxicity and enhances longevity by an hsf-1-dependent mechanism in *Caenorhabditis elegans*. Aging Cell.

[153] Gülçin I., Elmastaş M., Aboul-Enein H.Y. (2012). Antioxidant activity of clove oil–A powerful antioxidant source. Arab. J. Chem..

[154] Sadeli R.A. (2016). Uji Aktivitas Antioksidan Dengan Metode DPPH (1, 1-Diphenyl-2-Picrylhydrazyl) Ekstrak Bromelain Buah Nanas (*Ananas comosus* (L.) Merr.). Ph.D. Thesis.

[155] Rusmarilin H., Lubis Z., Lubis L.M., Barutu Y.A.P. (2019). Potential of natural antioxidants of black cumin seed (*Nigella sativa*) and sesame seed (*Sesamum indicum*) extract by microencapsulation methods. IOP Conf. Ser. Earth Environ. Sci. IOP Publ..

[156] Suhaling S. (2010). Uji Aktivitas Antioksidan Ekstrak Metanol Kacang Merah (*Phaseolus vulgaris* L.) Dengan Metode DPPH. Master’s Thesis.

[157] Ikhlas N. Uji Aktivitas Antioksidan Ekstrak Herba Kemangi (*Ocimum americanum* Linn) dengan Metode DPPH (2, 2-Difenil-1-Pikrilhidrazil). Master’s Thesis. 2013. http://repository.uinjkt.ac.id/dspace/handle/123456789/25905.

[158] Chae H.S., Park H.J., Hwang H.R., Kwon A., Lim W.H., Yi W.J., Han D.H., Kim Y.H., Baek J.H. (2011). The effect of antioxidants on the production of pro-inflammatory cytokines and orthodontic tooth movement. Mol. Cells.

[159] Nau R., Eiffert H. (2002). Modulation of release of proinflammatory bacterial compounds by antibacterials: Potential impact on course of inflammation and outcome in sepsis and meningitis. Clin. Microbiol. Rev..

[160] Luderitz O., Tanamoto K., Galanos C., McKenzie G.R., Brade H., Zahringer U., Rietschel E.T., Kusumoto S., Shiba T. (1984). Lipopolysaccharides: Structural principles and biologic activities. Clin. Infect. Dis..

[161] Sejvar J., Lutterloh E., Naiene J., Likaka A., Manda R., Nygren B., Monroe S., Khaila T., Lowther S.A., Capewell L. (2012). Neurologic manifestations associated with an outbreak of typhoid fever, Malawi—Mozambique, 2009: An epidemiologic investigation. PLoS ONE.

[162] Alvarez-Arellano L., Maldonado-Bernal C. (2014). *Helicobacter pylori* and neurological diseases: Married by the laws of inflammation. World J. Gastrointest Pathophysiol..

[163] Arneth B.M. (2018). Gut–brain axis biochemical signalling from the gastrointestinal tract to the central nervous system: Gut dysbiosis and altered brain function. Postgrad. Med. J..

[164] Sabetghadam A., Ramanathan S., Sasidharan S., Mansor S.M. (2013). Subchronic exposure to mitragynine, the principal alkaloid of *Mitragyna speciosa*, in rats. J. Ethnopharmacol..

[165] Chittrakarn S., Sawangjaroen K., Prasettho S., Janchawee B., Keawpradub N. (2008). Inhibitory effects of kratom leaf extract (*Mitragyna speciosa* Korth.) on the rat gastrointestinal tract. J. Ethnopharmacol..

[166] Tsuchiya S., Miyashita S., Yamamoto M., Horie S., Sakai S.-I., Aimi N., Takayama H., Watanabe K. (2002). Effect of mitragynine, derived from Thai folk medicine, on gastric acid secretion through opioid receptor in anesthetized rats. Eur. J. Pharmacol..

[167] Anwar M., Law R., Schier J. (2016). Notes from the field: Kratom (*Mitragyna speciosa*) exposures reported to poison centers—United States, 2010–2015. Morb. Mortal. Wkly. Rep..

[168] Scott Gottlieb M.D. Agency’s Scientific Evidence on the Presence of Opioid Compounds in Kratom, Underscoring Its Potential for Abuse, Food and Drug Administration Statement from FDA Commissioner. Statement from FDA Commissioner, [Press Release] 6 February 2018. https://www.fda.gov/NewsEvents/Newsroom/PressAnnouncements/ucm595622.htm.

[169] Sethi R., Hoang N., Ravishankar D.A., McCracken M., Manzardo A.M. (2020). Kratom (*Mitragyna speciosa*): Friend or foe?. Prim. Care Companion CNS Disord..

[170] Griffiths C.L., Gandhi N., Olin J.L. (2018). Possible kratom-induced hepatomegaly: A case report. J. Am. Pharm. Assoc..

[171] Tayabali K., Bolzon C., Foster P., Patel J., Kalim M.O. (2018). Kratom: A dangerous player in the opioid crisis. J. Community Hosp. Intern. Med. Perspect.

[172] Chittrakarn S., Keawpradub N., Sawangjaroen K., Kansenalak S., Janchawee B. (2010). The neuromuscular blockade produced by pure alkaloid, mitragynine and methanol extract of kratom leaves (*Mitragyna speciosa* Korth. ). J. Ethnopharmacol..

[173] Hanapi N., Azizi J., Ismail S., Mansor S. (2010). Evaluation of Selected Malaysian Medicinal Plants on Phase I Drug Metabolizing Enzymes, CYP 2 C 9, CYP 2 D 6 and CYP 3 A 4 Activities in vitro. Int. J. Pharmacol..

[174] Purintrapiban J., Keawpradub N., Kansenalak S., Chittrakarn S., Janchawee B., Sawangjaroen K. (2011). Study on glucose transport in muscle cells by extracts from *Mitragyna speciosa* (Korth) and mitragynine. Nat. Prod. Res..

[175] Nelsen J.L., Lapoint J., Hodgman M.J., Aldous K.M. (2010). Seizure and coma following Kratom (*Mitragynina speciosa* Korth) exposure. J. Med. Toxicol..

[176] Kapp F.G., Maurer H.H., Auwärter V., Winkelmann M., Hermanns-Clausen M. (2011). Intrahepatic cholestasis following abuse of powdered kratom (*Mitragyna speciosa*). J. Med. Toxicol..

[177] Dorman C., Wong M., Khan A. (2015). Cholestatic hepatitis from prolonged kratom use: A case report. Hepatology.

[178] Riverso M., Chang M., Soldevila-Pico C., Lai J., Liu X. (2018). Histologic Characterization of Kratom Use-Associated Liver Injury. Gastroenterol. Res..

[179] Fernandes C.T., Iqbal U., Tighe S.P., Ahmed A. (2019). Kratom-Induced Cholestatic Liver Injury and Its Conservative Management. J. Investig. Med. High Impact Case Rep..

[180] Palasamudram Shekar S., Rojas E.E., D’Angelo C.C., Gillenwater S.R., Galvis N.P.M. (2019). Legally Lethal Kratom: A Herbal Supplement with Overdose Potential. J. Psychoact. Drugs.

[181] Mousa M.S., Sephien A., Gutierrez J., O’Leary C. (2018). N-Acetylcysteine for Acute Hepatitis Induced by Kratom Herbal Tea. Am. J. Ther..

[182] Osborne C.S., Overstreet A.N., Rockey D.C., Schreiner A.D. (2019). Drug-Induced Liver Injury Caused by Kratom Use as an Alternative Pain Treatment Amid an Ongoing Opioid Epidemic. J. Investig. Med. High Impact Case Rep..

